# DOT1L Drives Endothelial‐to‐Mesenchymal Transition and Fibrotic Vascular Remodeling via H3K79 Methylation

**DOI:** 10.1002/advs.202515581

**Published:** 2026-04-03

**Authors:** Yaofeng Wang, Xing Peng, Jingjing Chen, Yun Zhang, Tinghong Zhang, Jingyuan Zhang, Jiaying Fan, Hui Zheng, Qiaoyuan Liu, Zhimin Song, Zhan‐Peng Huang, Shu Meng

**Affiliations:** ^1^ Department of Basic Science Research Guangzhou National Laboratory Guangzhou Guangdong China; ^2^ Zhongshan School of Medicine Sun Yat‐sen University Guangzhou Guangdong China; ^3^ State Key Laboratory of Respiratory Disease The First Affiliated Hospital Guangzhou Medical University Guangzhou Guangdong China; ^4^ Department of Cardiology Center for Translational Medicine of Precision Medicine The First Affiliated Hospital Sun Yat‐sen University Guangzhou Guangdong China; ^5^ NHC Key Laboratory of Assisted Circulation Sun Yat‐sen University Guangzhou Guangdong China

**Keywords:** Dot1L, EndoMT, epigenetic regulation, pulmonary fibrosis

## Abstract

The lung is a highly vascularized organ in which endothelial cells (ECs) play a pivotal role in maintaining tissue homeostasis and regulating gas‐blood exchange. Increasing evidence suggests that endothelial‐to‐mesenchymal transition (EndoMT) contributes to fibrosis; however, the underlying epigenetic mechanisms remain incompletely understood. Here, we identify disruptor of telomeric silencing 1‐like (DOT1L), a histone H3 lysine 79 (H3K79) methyltransferase, as a key epigenetic regulator of EndoMT and fibrotic progression. In human umbilical vein ECs, TGFβ stimulation upregulated DOT1L expression and increased H3K79me2 levels during EndoMT. DOT1L knockdown abrogated H3K79 methylation and suppressed the expression of fibrosis‐associated genes. Chromatin immunoprecipitation analysis revealed that direct binding of SMAD2 to the DOT1L promoter increased its transcription and promoted H3K79me2 deposition at fibrosis‐related gene loci following TGFβ2 stimulation. In vivo, endothelial lineage‐tracing in mice demonstrated H3K79me2 accumulation in ECs undergoing EndoMT during bleomycin‐induced pulmonary fibrosis. Importantly, endothelial‐specific deletion of Dot1L significantly attenuated fibrotic remodeling, collagen deposition, and mesenchymal marker expression. Collectively, these findings establish DOT1L as a critical epigenetic driver of EndoMT and pulmonary fibrosis through H3K79me2‐mediated transcriptional activation, highlighting it as a potential therapeutic target in fibrotic lung disease.

## Graphical abstract

DOT1L as a central epigenetic regulator of EndoMT and pulmonary fibrosis. Acting as an early epigenetic switch, it translates TGFβ–SMAD signaling into H3K79me2‐mediated chromatin remodeling, selectively activates fibrosis‐related genes, and primes ECs for rapid mesenchymal transition. These findings illuminate the molecular underpinnings of EndoMT and position DOT1L as a compelling therapeutic target in IPF and other fibrotic diseases.

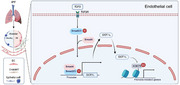

## 1 Introduction

Transdifferentiation is the direct conversion of one differentiated cell type into another [1]. While this phenomenon is commonly observed in lower vertebrates and in mammals during embryonic development, it is rare in adult tissues. A notable exception is endothelial‐to‐mesenchymal transition (EndoMT), one of the most prevalent forms of transdifferentiation in adult mammals, in which vascular endothelial cells (ECs) acquire mesenchymal and fibroblast‐like phenotypes.

EndoMT has been implicated in a wide range of pathological conditions, including atherosclerosis [2, 3], cerebral cavernous malformation [4], pulmonary hypertension [5], and a variety of organ fibrosis [6, 7, 8, 9]. During this process, ECs undergo profound phenotypic, functional, and transcriptomic reprogramming, characterized by loss of canonical endothelial markers and acquisition of mesenchymal and fibrotic features [10]. However, a fundamental question remains unresolved: how is epigenetic plasticity regulated to permit such a rapid and profound cell fate transition?

Epigenetic modifications such as DNA methylation and histone modifications play essential roles in orchestrating gene expression without altering the underlying DNA sequence [11]. Recent studies have implicated epigenetic regulation in EndoMT, modulating the expression of genes critical for this process [12, 13, 14]. However, a comprehensive understanding of how epigenetic mechanisms govern the EndoMT transcriptional landscape remains elusive.

Accumulating evidence suggests that transdifferentiation can be experimentally induced by the forced overexpression of lineage‐specific transcription factors or growth factors [15, 16, 17]. Many pathological stimuli, such as transforming growth factor beta (TGFβ), hypoxia, and proinflammatory cytokines, promote the EndoMT process in vitro and in vivo [10, 18, 19]. TGFβ is a key driver, particularly in the context of pulmonary fibrosis [20, 21], where EndoMT has been shown to directly contribute to pulmonary fibrosis using endothelial lineage‐tracing mice [9]. Despite these insights, the epigenetic regulators mediating TGFβ‐driven EndoMT remain largely undefined.

Here, we identify disruptor of telomeric silencing 1‐like (DOT1L), the sole H3K79 methyltransferase, as a key epigenetic regulator of TGFβ‐induced EndoMT. A targeted screen revealed DOT1L as the only epigenetic modifier upregulated early in the transition, and its depletion abolished H3K79me2 and suppressed EndoMT. We further demonstrate that TGFβ–SMAD signaling induces DOT1L, which drives fibrogenic gene activation through H3K79me2 deposition. Using endothelial‐specific Dot1L knockout mice and lineage tracing, we demonstrate that DOT1L mediates EndoMT and pulmonary fibrosis In vivo, establishing DOT1L as a critical epigenetic driver of endothelial plasticity.

## 2 Results

### 2.1 DOT1L Upregulation Mediates TGFβ‐Induced EndoMT

EndoMT, the direct conversion of ECs into fibroblasts, represents a rapid and tightly regulated cell fate switch. This transition involves the prompt activation of a broad repertoire of fibroblast‐associated genes that are typically epigenetically silenced in ECs, suggesting that EndoMT requires extensive epigenetic reprogramming, particularly the establishment of permissive chromatin states at previously repressed loci.

**FIGURE 1:**
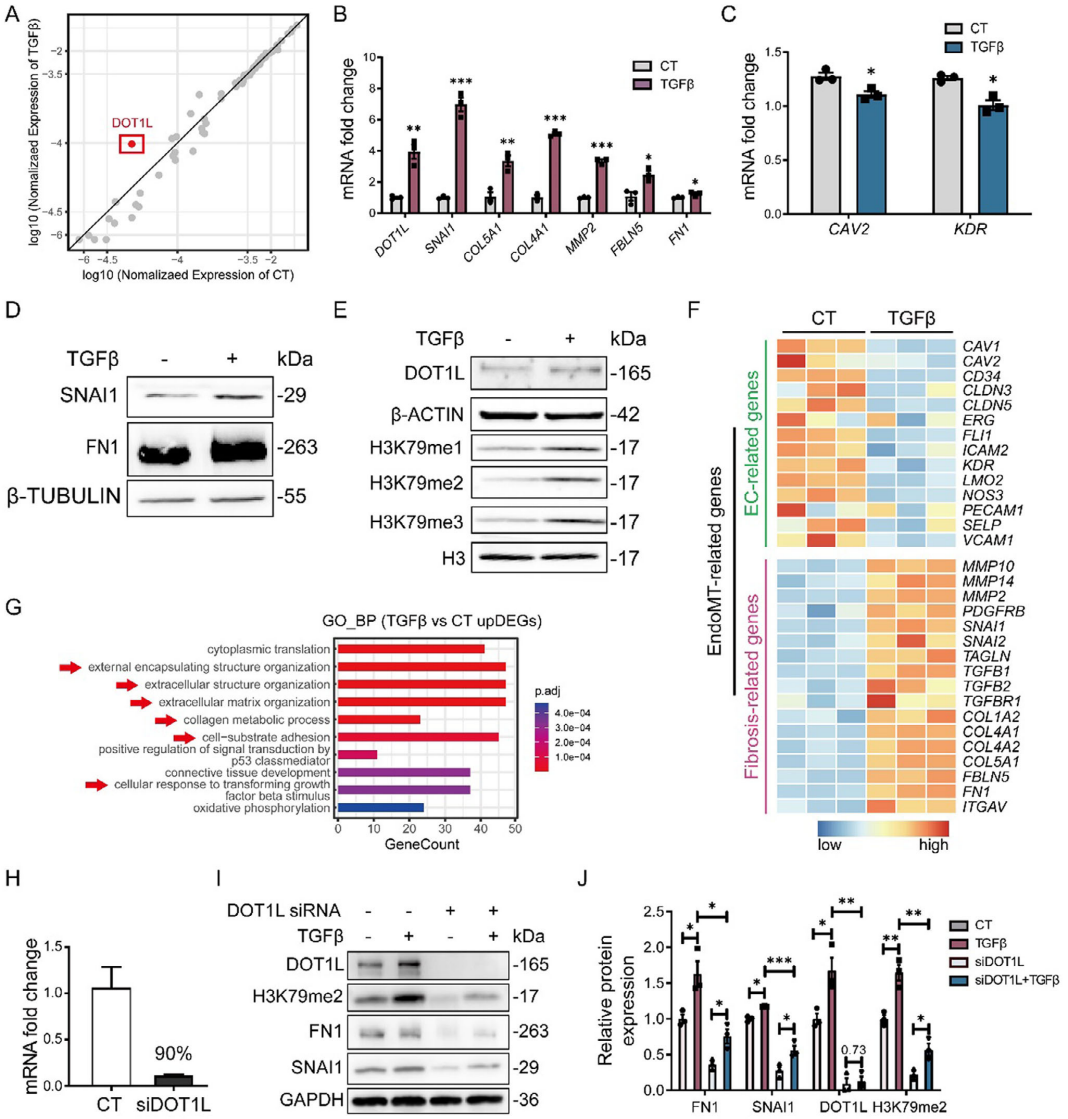
DOT1L upregulation mediates TGFβ‐induced EndoMT. (A‐G) HUVECs were treated with 10 ng/ml TGFβ2 for 24 h (*n* = 3). (A) RT‐qPCR array of 84 epigenetic modifiers. (B,C). RT‐qPCR analysis of *DOT1L* and mesenchymal markers (*COL5A1*, *MMP2*, *FBLN5*, *FN1*) and endothelial markers (*CAV2*, *KDR*). (D,E) Western blot of mesenchymal markers (SNAI1, FN1), and DOT1L, H3K79me1, H3K79me2, H3K79me3. (F) Heatmap of EndoMT‐related gene expression. (G) GO enrichment analysis of DEGs in RNA‐seq. Red arrows highlight fibrosis‐related pathways. (H‐J) HUVECs were transfected with CT or DOT1L siRNA for 48 h, followed by 10 ng/ml TGFβ2 treatment for 24 h (*n* = 3). (H). RT‐qPCR analysis of DOT1L expression. (I) Western blot of DOT1L, H3K79me2, and mesenchymal markers (SNAI1, FN1). (J) Quantification of I. Data are presented as mean± S.E.M. ^*^ *p* < 0.05, ^**^ *p* < 0.01, ^***^ *p* < 0.001.

TGFβ is a well‐established inducer of EndoMT and tissue fibrosis [22, 23, 24]. We hypothesized that in TGFβ‐induced EndoMT, specific epigenetic modifiers are upregulated to catalyze the deposition of active histone marks on pro‐fibrotic genes, thereby facilitating their transcriptional activation. To test this, we conducted a focused screen of 84 epigenetic regulators using an RT‐qPCR Array of Human Epigenetic Chromatin Modification Enzymes in HUVECs treated with or without TGFβ2 for 24 h. Strikingly, among the screened factors, only one gene, DOT1L, was upregulated by more than twofold (Figure 1A).

We next investigated the temporal dynamics of TGFβ‐induced EndoMT. Consistent with previous reports, EndoMT was most prominently induced at day 7, as evidenced by robust upregulation of mesenchymal markers *SNAI1* and *SNAI2* (Figure S1A) and concurrent downregulation of endothelial markers *PECAM1* and *CDH5* (Figure S1B) at the mRNA level. Notably, *DOT1L* gene expression peaked at 24 h and returned to baseline at day 7 (Figure S1C), suggesting that *DOT1L* is an early‐responsive gene of TGFβ signaling.

Given that DOT1L is the sole known methyltransferase responsible for H3K79 methylation, we next assessed whether its induction increases H3K79 methylation. As expected, TGFβ stimulation resulted in robust upregulation of DOT1L at both mRNA and protein levels, accompanied by a progressive increase in H3K79 mono‐, di‐, and tri‐methylation (Figure 1B–E).

Based on these observations, we selected 24 h of TGFβ2 treatment as the standard condition for subsequent experiments. RT‐qPCR analysis confirmed upregulation of fibrosis‐related genes (Figure 1B) and downregulation of endothelial‐specific genes (Figure 1C), findings that were corroborated at the protein level (Figure 1D). Increased DOT1L protein expression was accompanied by elevated H3K79 methylation across all forms (Figure 1E).

To validate these findings at a transcriptomic level, we performed bulk RNA‐seq in HUVECs treated with or without TGFβ2 for 24 h. Spearman correlation analysis confirmed strong reproducibility across biological replicates (Figure S1D). Heatmap analysis of differentially expressed genes (DEGs) revealed a coordinated transcriptional shift, characterized by downregulation of endothelial genes and upregulation of fibrosis‐associated genes (Figure 1F). GO analysis revealed that DEGs were highly enriched in fibrosis‐associated pathways, including extracellular organization, cell‐substrate adhesion, and cellular response to transforming growth factor beta (Figure 1G). These data suggest that DOT1L and H3K79 methylation are specifically upregulated in TGFβ‐induced EndoMT.

To examine whether DOT1L mediates EndoMT, we performed DOT1L siRNA knockdown in HUVECs. DOT1L mRNA levels were reduced by ∼90% (Figure 1H), and the western blot confirmed effective protein depletion (Figure 1I). Notably, DOT1L knockdown abolished the TGFβ2‐induced increase in H3K79me2 and markedly attenuated the induction of mesenchymal markers such as *FN1* and *SNAI1* (Figure 1I,J).

Together, these findings indicate that TGFβ2 promotes DOT1L expression and H3K79me2 deposition, which, in turn, mediate the transcriptional activation of fibrotic genes during EndoMT.

### 2.2 TGFβ2 Induces DOT1L Transcription through Canonical SMAD Signaling

We hypothesized that TGFβ2 induces DOT1L transcription via the canonical SMAD signaling pathway. TGFβ2 typically activates this pathway by binding to the TGFβ receptor, leading to the phosphorylation of SMAD2 and SMAD3. These phosphorylated SMADs then form a complex with SMAD4 and translocate to the nucleus to activate the expression of target genes [21]. To investigate whether this pathway is involved in TGFβ2‐induced DOT1L expression, we analyzed the temporal dynamics of SMAD activation in ECs during EndoMT.

**FIGURE 2:**
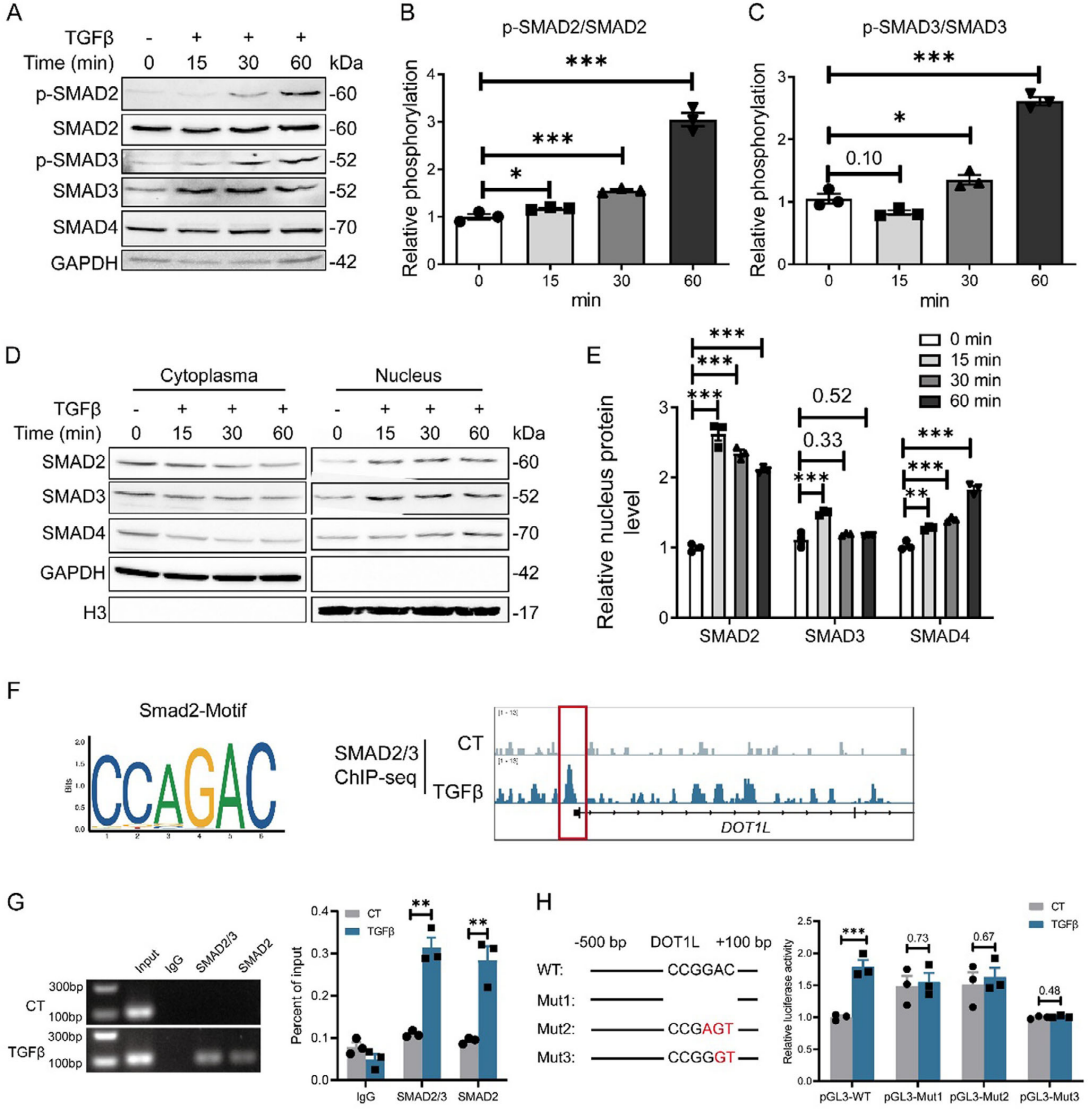
TGFβ2 induces DOT1L transcription through canonical SMAD signaling. (A‐E) HUVECs were treated with 10 ng/ml TGFβ2 for 0, 15, 30, and 60 min (n = 3). (A) Western blot of SMAD2, SMAD3, and SMAD4 phosphorylation. (B) Relative SMAD2 phosphorylation. (C) Relative SMAD3 phosphorylation. (D) Western blot of SMAD2, SMAD3, and SMAD4 in cytoplasm and nucleus. (E) Relative nuclear SMAD2, SMAD3, and SMAD4 expression. (F‐G) HUVECs were treated with 10 ng/ml TGFβ2 for 24 h. (F) ChIP‐seq of SMAD2/3 binding at the DOT1L promoter. (G) ChIP‐PCR of SMAD2/3 and SMAD2 at DOT1L promoter (n = 3). (H) Luciferase activity in TIME cells transfected with wild‐type or mutant DOT1L promoter plasmid for 24 h, followed by TGFβ2 treatment for 4 h (n = 3). Data are presented as mean± S.E.M. ^*^ *p* < 0.05, ^**^ *p* < 0.01, ^***^ *p* < 0.001.

Western blot analysis revealed a time‐dependent increase in SMAD2 and SMAD3 phosphorylation, with elevated levels observed at 15, 30, and 60 min following TGFβ2 treatment (Figure 2A–C). Subcellular fractionation further confirmed translocation of SMAD2, SMAD3, and SMAD4 from the cytosol to the nucleus in response to TGFβ2 stimulation (Figure 2D,E).

We then performed a transcription factor binding prediction of SMAD in DOT1L promoter. Using the JASPAR database, we identified a SMAD2 binding motif near the DOT1L transcription start site (TSS). To test this hypothesis, we performed ChIP‐seq using anti‐SMAD2/3 antibodies and observed a significant increase in SMAD2/3 binding at the DOT1L promoter upon TGFβ2 stimulation, particularly within the predicted SMAD binding region (highlighted by the red box in Figure 2F). This result was further validated by ChIP‐PCR with an additional SMAD2 antibody using primers surrounding the binding site (Figure 2G).

To investigate whether SMAD2 binding is required for DOT1L transcriptional activation, we generated pGL3‐luciferase reporter constructs containing the DOT1L wild‐type promoter (−500 bp to +100 bp), which encompasses the SMAD2 binding site (Figure 2H), as well as promoter mutants in which the SMAD2 binding site was deleted or mutated. As expected, TGFβ2 treatment significantly increased luciferase activity in cells transfected with the wild‐type promoter, whereas this induction was abolished in all mutant constructs (Figure 2H). These findings indicate that TGFβ2 induces DOT1L transcription through SMAD2 binding to its promoter.

### 2.3 TGFβ2 Enhances H3K79me2 Occupancy at Fibrosis‐Associated Gene Bodies and Promotes Their Transcription during EndoMT

Given that DOT1L is the only identified methyltransferase responsible for H3K79 methylation, and that H3K79me2 is associated with active transcription [25, 26, 27], we hypothesized that DOT1L promotes EndoMT through the deposition of H3K79me2 marks on fibrosis‐associated genes, thereby activating their transcription.

**FIGURE 3:**
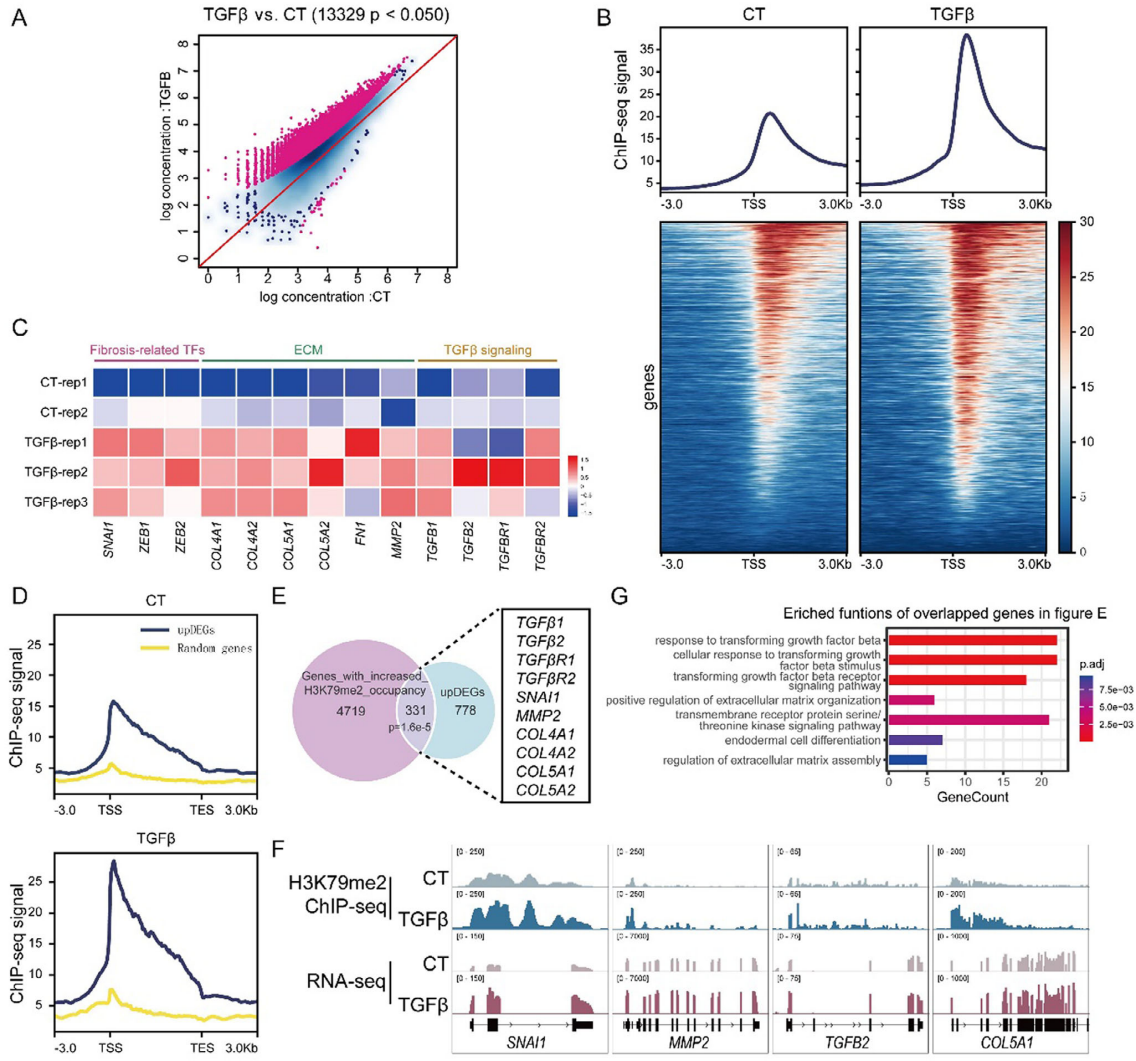
TGFβ2 enhances H3K79me2 occupancy in the gene bodies of fibrosis‐related genes and upregulates their transcription during EndoMT in vitro. HUVEC were treated with 10 ng/ml TGFβ2 for 24 h. (A) Differential H3K79me2 binding peaks between CT and TGFβ2‐treated cells in ChIP‐seq by dot plot. (B) MA plot and heatmap of H3K79me2 ChIP‐seq in all different peak genes, using a 3 kb window centered on TSS regions. (C) Heatmap of H3K79me2 ChIP‐seq showing signals of fibrosis‐related genes. (D) MA plot of H3K79me2 ChIP‐seq in 1k random genes and upregulated DEGs in RNA‐seq with a −3 kb to +3 kb window around the gene body. (E) Venn diagram showing 331 overlapping genes with increased H3K79me2 occupancy in H3K79me2 ChIP‐seq and upregulated DEGs in RNA‐seq. (F) ChIP‐seq and RNA‐seq signal at the indicated genomic locus. (G) GO analysis of the overlapping genes in E. Data are presented as mean± S.E.M.

To test this hypothesis, we performed ChIP‐seq analysis for H3K79me2 in HUVECs treated with or without TGFβ2 for 24 h, and integrated these data with RNA‐seq profiles. Principal component analysis confirmed high reproducibility of these datasets (Figure S2A), with the majority of H3K79me2 peaks mapped to protein‐coding genes (Figure S2B). Genome‐wide mapping revealed that H3K79me2 peaks were broadly distributed across gene bodies, with preferential localization in intronic regions and a gradual decrease toward the 3′ end (Figure 3B). TGFβ2 treatment significantly increased H3K79me2 peak intensity compared to control cells (Figure 3A,B).

We next focused on H3K79me2 enrichment at key fibrosis‐related genes, including *TGFB1*, *TGFB2*, *TGFBR1*, *TGFBR2*, *FN1*, *MMP2*, *COL4A1*, *COL4A2*, *COL5A1*, *COL5A2*, *SNAI1*, *ZEB1*, and *ZEB2*, which are associated with TGFβ signaling, extracellular matrix (ECM) remodeling, and fibrotic activation (Figure 3C; Figure S2C). TGFβ stimulation markedly increased H3K79me2 occupancy at these loci.

To assess the global impact of TGFβ on H3K79me2 distribution and transcriptional regulation, we integrated RNA‐seq and ChIP‐seq datasets. From RNA‐seq, we identified 1109 genes upregulated upon TGFβ stimulation. We then analyzed H3K79me2 occupancy across these genes using ChIP‐seq data and observed a pronounced genome‐wide increase following TGFβ treatment (Figure 3D), indicating a strong association between enhanced H3K79me2 deposition and transcriptional activation.

Further integrative analysis revealed 331 genes with both increased H3K79me2 and upregulated transcription following TGFβ2 treatment (Figure 3E). Notably, this subset included key fibrosis‐related genes such as *SNAI1*, *MMP2*, *TGFB2*, *TGFBR2*, and *COL4A1*, all of which showed concordant increases in H3K79me2 signals and mRNA expression (Figure 3F), confirmed by ChIP‐qPCR (Figure S2D) and RT‐qPCR (Figure 1B,C). Functional enrichment analysis of these genes highlighted significant involvement in EndoMT‐related pathways, including TGFβ signaling response and ECM organization (Figure 3G).

Together, these findings indicate that TGFβ2 induces H3K79me2 deposition at fibrosis‐associated genes, driving their transcriptional upregulation and contributing to EndoMT in vitro.

### 2.4 DOT1L and H3K79me2 Levels Are Elevated in a Murine Pulmonary Fibrosis Model

**FIGURE 4:**
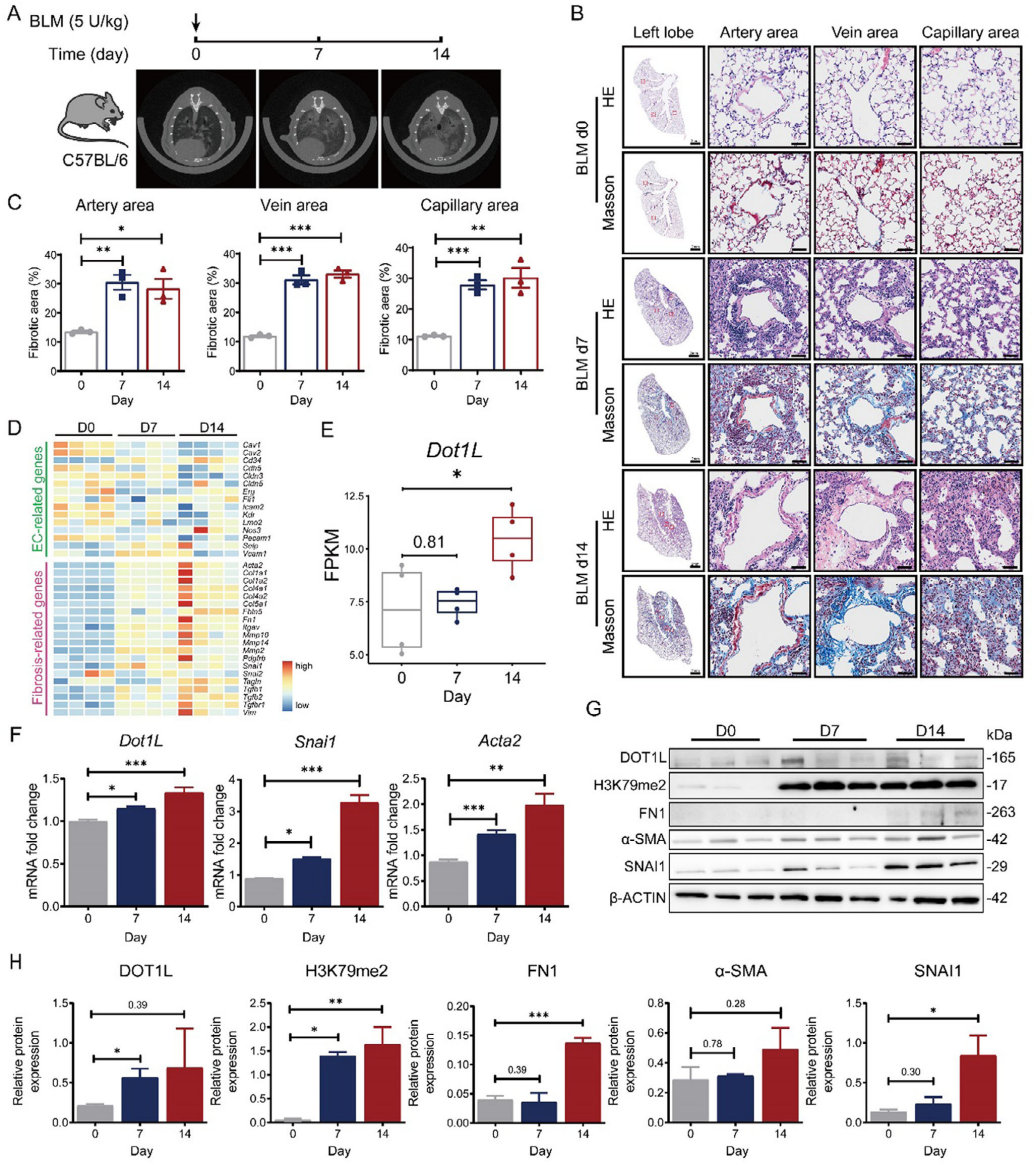
DOT1L and H3K79me2 levels are elevated in murine pulmonary fibrosis model. (A) Micro‐CT images of the mouse lungs. (B) H and E and Masson's trichrome staining of the left lung lobe, including artery, vein, and capillary areas. Scale bars: 2 mm (whole lung) and 50 µm (inset). (C) Quantification of Masson's trichrome staining in panel B. (D) Heatmap showing the expression of EndoMT‐related genes. (E) RNA‐seq analysis of Dot1L expression levels. (F) RT‐qPCR analysis showing the gene expression of Dot1L, Snai1, and Acta2. (G) Western blot analysis of DOT1L, H3K79me2, SNAI1, FN1, and α‐SMA protein levels. (H) Quantification of Western blot data in panel G. Data are presented as mean± S.E.M. ^*^ *p* < 0.05, ^**^ *p* < 0.01, ^***^ *p* < 0.001.

To assess the role of DOT1L and H3K79me2 in EndoMT and pulmonary fibrosis in vivo, we used a BLM‐induced pulmonary fibrosis model that replicates human IPF pathology [28]. A single intratracheal dose of 5 U/kg BLM was administered to induce fibrosis. Progressive fibrosis was confirmed by micro‐CT imaging and histological analysis (Figure 4A–C). Fibrotic changes were most prominent in perivascular regions, as shown by H and E and Masson's trichrome staining (Figure 4B). Quantification of collagen deposition revealed a marked increase in fibrosis around arteries, veins, and capillaries (Figure 4B,C), indicating a spatial link between vascular structures and fibrotic remodeling.

To systematically characterize transcriptomic alterations during fibrosis progression, we performed bulk RNA‐seq on murine lung tissues collected at days 0, 7, and 14 post‐injury. Principal component analysis showed clear clustering and high reproducibility among biological replicates (Figure S3A). Heatmap profiling of DEGs showed a gene signature consistent with EndoMT, marked by a coordinated downregulation of endothelial‐specific genes and an upregulation of fibrosis‐associated genes (Figure 4D). Gene set enrichment analysis (GSEA) further demonstrated that DEGs were highly enriched in gene sets related to lung fibrosis (Figure S3B,C), and were strongly associated with biological processes such as ECM organization, cell–substrate adhesion, and wound healing (Figure S3D,E).

Transcript‐level analysis (FPKM) revealed a significant upregulation of *Dot1L* expression by day 14 compared to baseline (day 0) (Figure 4E). These findings were confirmed by RT–PCR, which showed elevated expression of *Dot1L, Snai1*, and *Acta2* at both day 7 and 14 (Figure 4F). At the protein level, DOT1L expression was markedly increased at day 7, while H3K79me2 levels remained persistently elevated throughout the fibrotic phase (Figure 4G,H). In parallel, protein levels of SNAI1 and FN1 were significantly increased at day 14, and α‐SMA exhibited a trend toward upregulation (Figure 4 G,H).

### 2.5 EndoMT Contributes to Pulmonary Fibrosis

**FIGURE 5:**
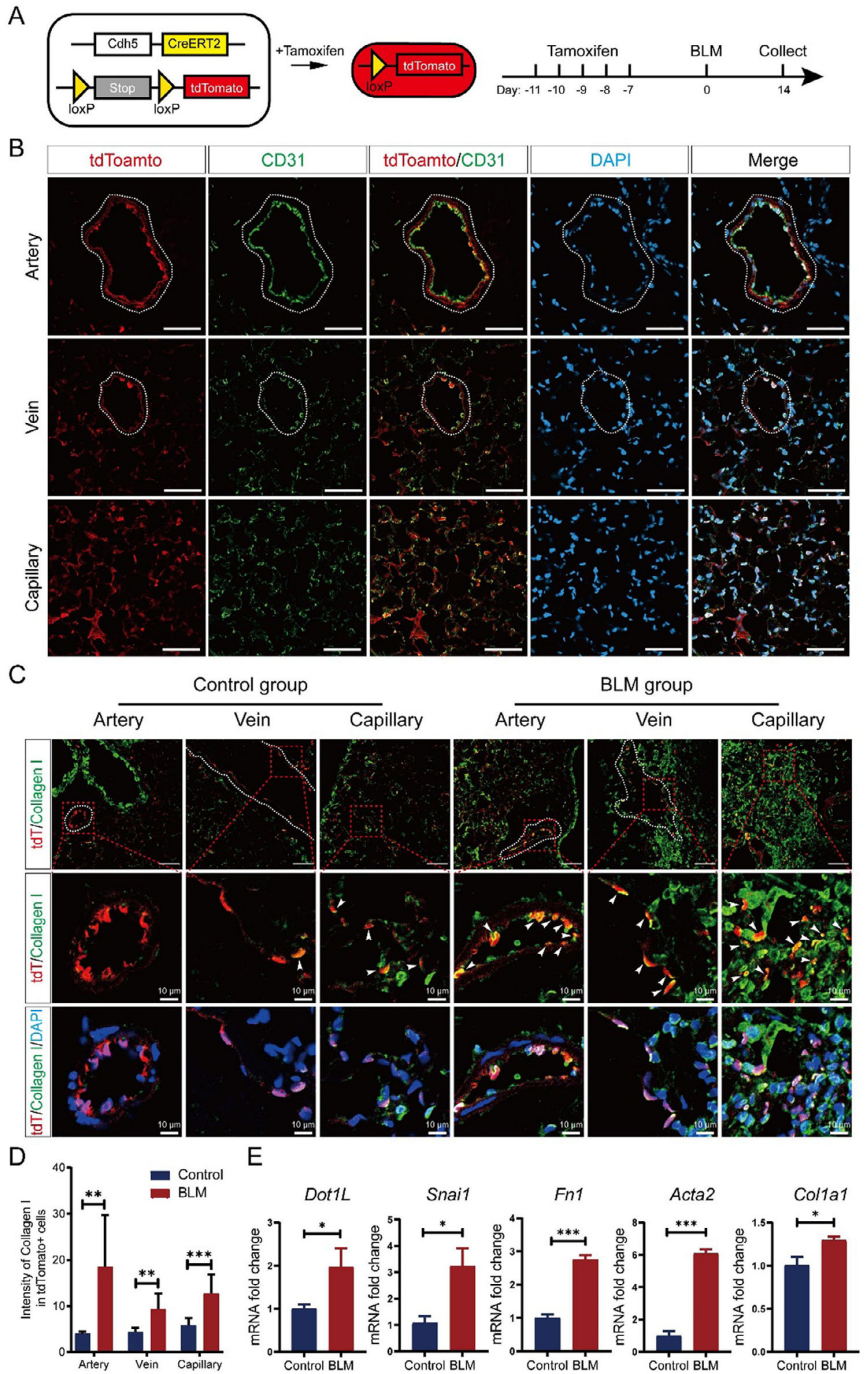
EndoMT contributes to pulmonary fibrosis. (A) Schematic of the Cdh5‐creERT2; Rosa26‐tdTomato mouse experiment. (B) IF showing the staining of CD31 (PECAM1) in Cdh5‐creERT2; Rosa26‐tdTomato mice. Scale bar: 50 µm. (C) IF showing the staining of Collagen I in day 0 and 14 in Cdh5‐creERT2; Rosa26‐tdTomato mice. Scale bars: 50 µm and 10 µm. (D) Collagen I intensity in tdTomato‐positive ECs. (E) RT‐qPCR analysis showing the gene expression of *Dot1L*, *Acta2*, *Snai1*, *Col1a1*, and *Fn1* in isolated mouse lung ECs from day 0 and day 7 post‐BLM treatment. Data are presented as mean± S.E.M. ^*^ *p* < 0.05, ^**^ *p* < 0.01, ^***^ *p* < 0.001.

Previous studies using the endothelial lineage‐tracing mouse model (Tie2‐Cre) suggest EndoMT occurs in IPF [9]; however, Tie2‐Cre lacks endothelial specificity, complicating the interpretation of lineage origin. To further validate the occurrence of EndoMT during pulmonary fibrosis, we utilized an endothelial lineage‐tracing system by crossing Cdh5‐CreERT2 mice with Rosa26‐tdTomato reporter mice (Figure 5A). Seven days following intraperitoneal administration of tamoxifen (100 mg/kg daily for 5 consecutive days), FACS analysis of digested lung tissue confirmed that tdTomato^+^ cells were restricted to the CD31^+^ endothelial population, comprising approximately 60% of CD31^+^ cells (Figure S4). Immunofluorescence staining of lung sections further showed tdTomato signal restricted to the endothelium of arteries, veins, and capillaries, indicating efficient and specific EC labeling (Figure 5B).

**FIGURE 6:**
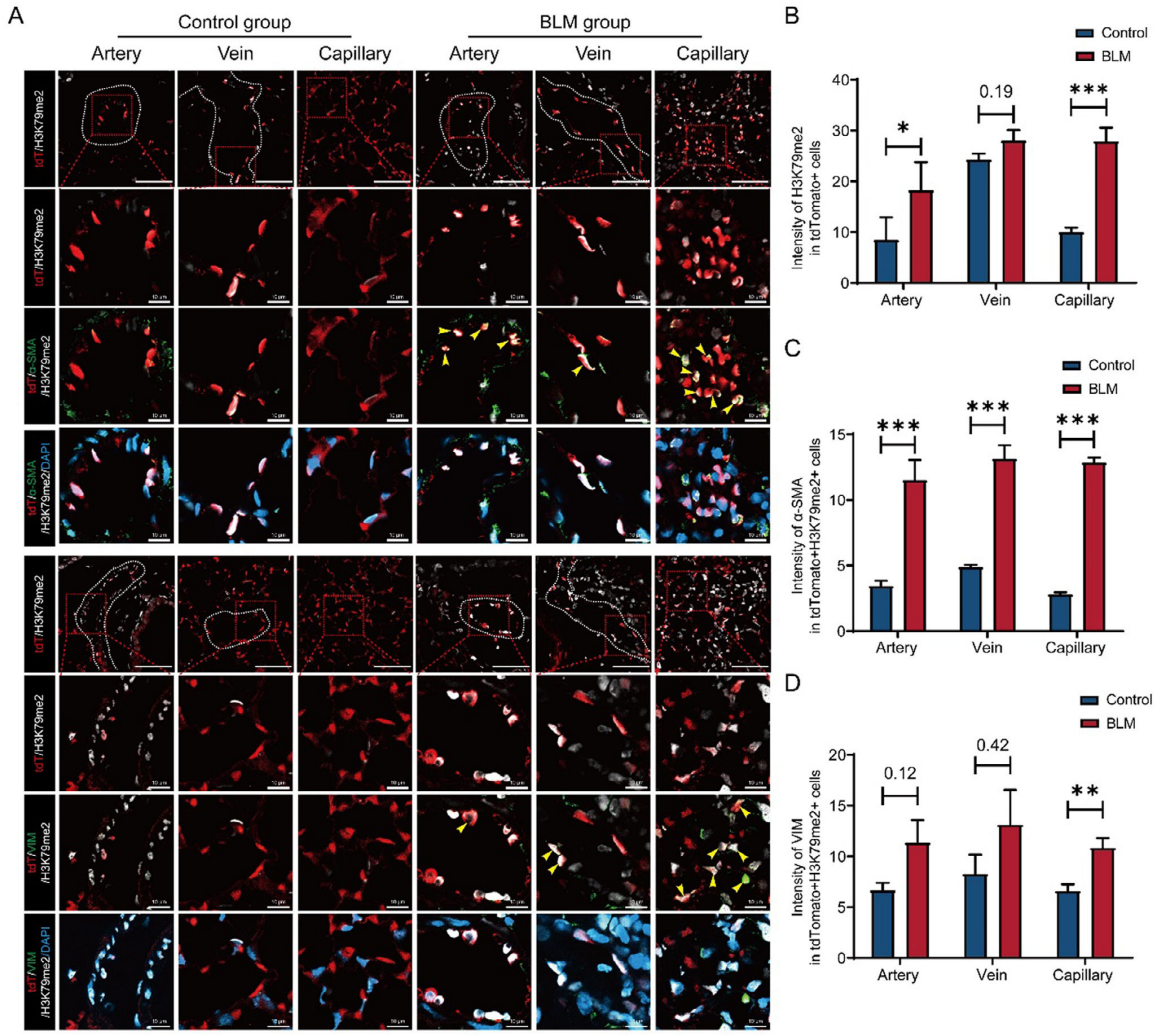
Endothelial H3K79me2 upregulation is associated with increased fibrotic marker expression in vivo. (A) IF showing co‐staining of H3K79me2 with α‐SMA or Vimentin in lungs from Cdh5‐creERT2; Rosa26‐tdTomato mice at day 0 and day 14 post‐BLM. Scale bars: 50 µm and 10 µm. (B) H3K79me2 intensity in tdTomato^+^ ECs. (C) α‐SMA intensity in H3K79me2^+^tdTomato^+^ cells. (D) Vimentin intensity in H3K79me2^+^tdTomato^+^ cells. Data are presented as mean± S.E.M. ^*^ *p* < 0.05, ^**^ *p* < 0.01, ^***^ *p* < 0.001.

Because tdTomato expression is permanent, this system enables lineage tracking of ECs even after loss of endothelial identity. We therefore induced pulmonary fibrosis in lineage‐traced mice (Figure 5A) and examined fibrotic gene expression within tdTomato^+^ cells. Following BLM treatment, tdTomato^+^ cells lining arterial, venous, and capillary vessels exhibited elevated expression of Collagen I and α‐SMA, while Vimentin was only elevated in capillary vessels (Figures 5C,D and 6A). These findings confirm that vascular ECs are capable of transitioning into mesenchymal‐like cells via EndoMT in fibrotic lungs. Notably, capillary ECs appeared more susceptible to EndoMT than arterial or venous ECs.

To further confirm that EndoMT occurs during IPF development, we FACS‐sorted CD45^−^CD31^+^ ECs from lung tissues at days 0 and 7 post‐BLM treatment and performed RT‐qPCR analysis. Indeed, at this early time point, *Dot1L* expression is significantly upregulated, along with a group of fibrosis‐related genes, including *Acta2*, *Snai1*, *Col1a1*, and *Fn1* (Figure 5E).

### 2.6 Endothelial H3K79me2 Upregulation Is Associated with Increased Fibrotic Marker Expression in vivo

To investigate the role of H3K79me2 in EndoMT In vivo, we performed co‐IF staining for H3K79me2 along with α‐SMA (Figure 6A–C) or Vimentin (Figure 6A,B,D), and assessed colocalization within tdTomato^+^ EC‐derived cells. Importantly, H3K79me2 levels were markedly elevated in arterial and capillary tdTomato^+^ ECs following BLM treatment compared to controls (Figure 6A,B). Quantitative analysis further demonstrated that α‐SMA intensity was significantly elevated in H3K79me2^+^tdTomato^+^ cells (Figure 6C). Notably, Vimentin intensity was increased only in capillary H3K79me2^+^tdTomato^+^ cells (Figure 6D,E). Together, these findings indicate that elevated endothelial H3K79me2 is associated with increased expression of profibrotic markers, supporting a link between H3K79me2 and the EndoMT, particularly in capillary ECs.

### 2.7 DOT1L Expression Is Upregulated in Arterial ECs and Aerocytes in Human IPF

To assess DOT1L expression in human pulmonary fibrosis, we analyzed a publicly available scRNA‐seq dataset (GSE136831) [29] comprising 32 IPF and 28 control lung samples (Figure S5A,B). Pulmonary ECs were categorized into six subtypes based on established marker genes [30] [31]: aerocytes, general capillary ECs (gCap ECs), atrial ECs, pulmonary venous ECs, systemic venous ECs, and lymphatic ECs. Among these, DOT1L expression was markedly increased in atrial ECs and aerocytes in IPF lungs compared with controls (Figure S5A). Notably, fibrosis‐associated genes, including *COL4A1*, *COL4A2*, *FBLN5*, *FN1*, *ITGAV*, and *VIM* were concurrently upregulated in these subtypes (Figure S5B), suggesting that elevated DOT1L may promote endothelial reprogramming toward a pro‐fibrotic state.

Aerocytes are recently defined specialized capillary ECs involved in gas‐blood exchange [31]. To further assess H3K79me2 levels in aerocytes and gCap ECs, we performed co‐IF staining for H3K79me2 with CAR4 (aerocyte marker) or PLVAP (gCap marker) [32] in the murine fibrosis model. Consistent with our earlier findings, both aerocytes and gCap ECs exhibited increased H3K79me2 levels following BLM exposure (Figure S5C–F). Remarkably, aerocytes showed the most pronounced upregulation of H3K79me2.

Together, these results indicate that DOT1L is upregulated in atrial ECs and aerocytes in human IPF lungs, where it may contribute to EndoMT and fibrotic progression.

### 2.8 Endothelial‐Specific Dot1L Knockout Mitigates EndoMT and Pulmonary Fibrosis

**FIGURE 7:**
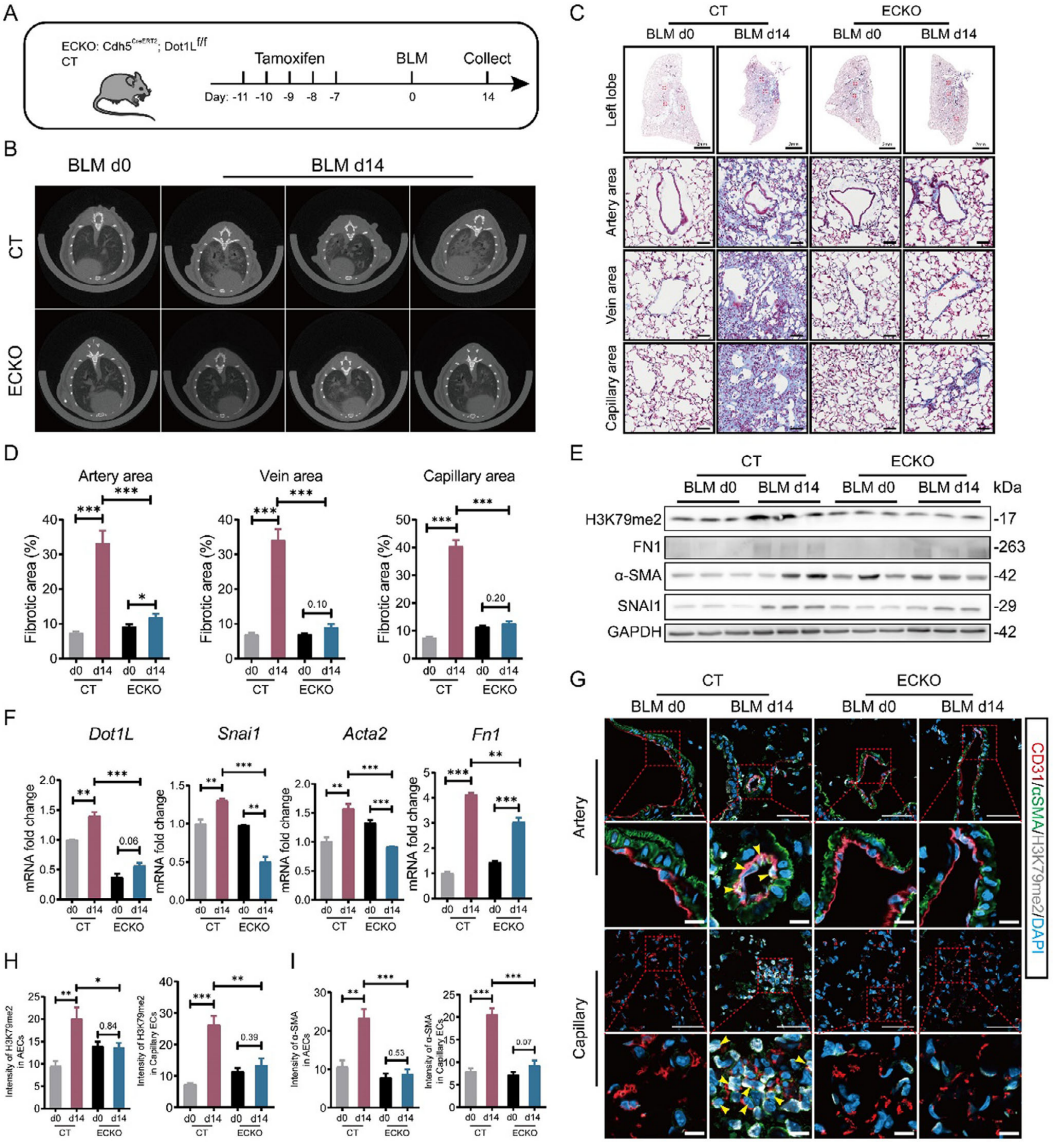
Endothelial‐specific Dot1L knockout mitigates EndoMT and pulmonary fibrosis. (A) Schematic experimental design. *n* = 9–10 mice in each group. (B) Micro‐CT images of lungs from control and BLM‐treated mice. (C) Masson's trichrome staining. Scale bars: 2 mm and 50 µm. (D) Quantification of Masson's trichrome staining. (E) Western blot showing expression of indicated proteins in lung tissues. (F) RT‐qPCR analysis of FACS‐sorted ECs. G. IF co‐staining of H3K79me2, α‐SMA, and CD31 at day 0 and day 14. Scale bar: 50 µm and 10 µm. H. H3K79me2 intensity in arterial ECs and capillary ECs. I. α‐SMA intensity in arterial ECs and capillary ECs. Data are presented as mean± S.E.M. ^*^ *p* < 0.05, ^**^ *p* < 0.01, ^***^ *p* < 0.001.

To investigate the functional role of DOT1L in EndoMT and pulmonary fibrosis in vivo, we generated EC‐specific Dot1L knockout mice (Dot1L‐ECKO; Cdh5‐CreERT2:Dot1L^f/f^) (Figure S6A,B). Pulmonary fibrosis was induced in Dot1L‐ECKO and littermate mice using BLM model (Figure 7A).

Compared to controls, Dot1L‐ECKO mice exhibited significantly reduced fibrosis as assessed by micro‐CT and histological analysis (Figure 7B–D; Figure S7A). Notably, fibrosis surrounding various vascular structures was largely absent in Dot1L‐ECKO lungs (Figure 7C,D), suggesting that endothelial DOT1L is critical for fibrotic vascular remodeling.

At the whole tissue level, the transcription upregulation of *Dot1L* and fibrosis‐related genes, including *Snai1*, *Fn1*, and *Col1a1*, observed in control mice following BLM treatment was largely blocked or attenuated in Dot1L‐ECKO mice. Conversely, the reduction of endothelial markers such as *Pecam1* and *Cdh5* in control mice was reversed in Dot1L‐ECKO lungs (Figure S7B). Accordingly, the protein levels of H3K79me2, SNAI1, and FN1, which were consistently upregulated in control mice, were markedly reduced in Dot1L‐ECKO mice (Figure 7E, Figure S7C).

To further confirm endothelial‐specific deletion, we FACS‐isolated CD45^−^CD31^+^ ECs from control and Dot1L‐ECKO mice at day 0 and day 14 post‐BLM and performed RT‐qPCR. *Dot1L* expression was drastically reduced in ECKO ECs, accompanied by corresponding decreases in fibrosis‐related transcripts (Figure 7F). Consistently, the induction of H3K79me2 and α‐SMA in arterial and capillary ECs after BLM treatment was blocked in Dot1L‐ECKO mice, as demonstrated by IF analyses (Figure 7G–I).

Together, these findings demonstrate that endothelial‐specific deletion of Dot1L attenuates collagen deposition and overall fibrotic severity. These data suggest that endothelial DOT1L promotes EndoMT and BLM‐induced pulmonary fibrosis through H3K79me2 deposition and transcriptional activation of profibrotic genes.

## 3 Discussion

In this study, we identified DOT1L as a critical epigenetic mediator of TGFβ‐induced EndoMT. Mechanistically, TGFβ2 upregulated DOT1L transcription via canonical SMAD signaling, with SMAD2 directly binding to the DOT1L promoter. Functionally, DOT1L promoted EndoMT by depositing H3K79me2 at the gene bodies of key fibrosis‐related loci, facilitating their transcriptional activation in vitro. Consistent with this, elevated DOT1L expression and H3K79me2 levels were observed in arterial and capillary ECs in human IPF samples and in murine fibrosis models, correlating with increased pro‐fibrotic gene expression. Endothelial‐specific knockout of Dot1L significantly attenuated EndoMT and pulmonary fibrosis, highlighting DOT1L as a promising therapeutic target for modulating EndoMT‐driven fibrotic remodeling.

Recent advances have highlighted the importance of epigenetic dysregulation in EndoMT [12, 13, 14] and fibrotic diseases [33]; however, the mechanisms underlying epigenetic plasticity during EndoMT remain incompletely understood. Through an unbiased screen of 84 epigenetic modifiers, we found that DOT1L is uniquely and rapidly upregulated at early time points. DOT1L participates in diverse biological processes, including telomeric silencing [34], transcriptional activation [26], transcriptional elongation [35], DNA damage repair [36] and cell cycle progression [37], and has been implicated in fibrotic remodeling across multiple organs, including peritoneal [38], cardiac [39], and renal fibrosis [40]. Although systemic pharmacological inhibition of DOT1L suppresses fibroblast activation and ameliorates pulmonary fibrosis [41], its epigenomic basis was unclear. Our integrative RNA‐seq and ChIP‐seq analyses reveal that DOT1L induces genome‐wide H3K79 methylation at fibrosis‐associated loci, promoting transcriptional activation and positioning DOT1L as a central driver of fibrotic remodeling across cell types.

IPF is a progressive and fatal interstitial lung disease of unknown etiology, characterized by persistent fibrotic remodeling, respiratory decline, and poor prognosis [42, 43]. Despite clinical use of antifibrotic agents, therapeutic efficacy remains modest [44, 45]. ECs, as the most abundant and functionally essential cell type for gas–blood exchange in the lung, have been underappreciated contributors to fibrosis. Previous work using Tie2‐Cre–based lineage tracing suggested that ECs undergo EndoMT [9]; here, using a more endothelial‐specific Cdh5‐CreERT2 lineage‐tracing system, we confirmed that EndoMT contributes directly to fibrotic vascular remodeling.

We observed that DOT1L expression is selectively elevated in aerocytes and arterial ECs in IPF patient. In the murine fibrosis model, we confirmed these patterns and found that capillary ECs are particularly susceptible to EndoMT, exhibiting elevated Dot1L expression, increased H3K79me2, and enhanced pro‐fibrotic signatures. Aerocytes are specialized for gas exchange, whereas gCap ECs maintain capillary integrity and repair [31]. EndoMT in these populations likely impairs endothelial function, exacerbating hypoxia and accelerating fibrosis. Notably, other epigenetic enzymes that may directly or indirectly modulate H3K79 methylation, including demethylase KDM2B [46] and E3 ubiquitin ligases of H2B [47, 48, 49, 50], remained largely unchanged in human and murine datasets, underscoring DOT1L as the primary driver of H3K79 methylation during EndoMT.

A defining feature of EndoMT is the rapid and coordinated transition from an endothelial to a mesenchymal transcriptional program, a scale of reprogramming that cannot be explained solely by transcription factor activation. Our findings support a model in which major EndoMT determinants such as TGFβ exert their earliest effects through the epigenome. Within hours of stimulation, SMAD2 is recruited to the DOT1L promoter, triggering swift induction of this histone methyltransferase. Once upregulated, DOT1L deposits H3K79me2 across pro‐fibrotic gene bodies, effectively opening chromatin and enabling high‐output transcriptional activation. Through this mechanism, TGFβ–SMAD signaling establishes an epigenetically permissive landscape that accelerates the endothelial fate switch.

**FIGURE 8:**
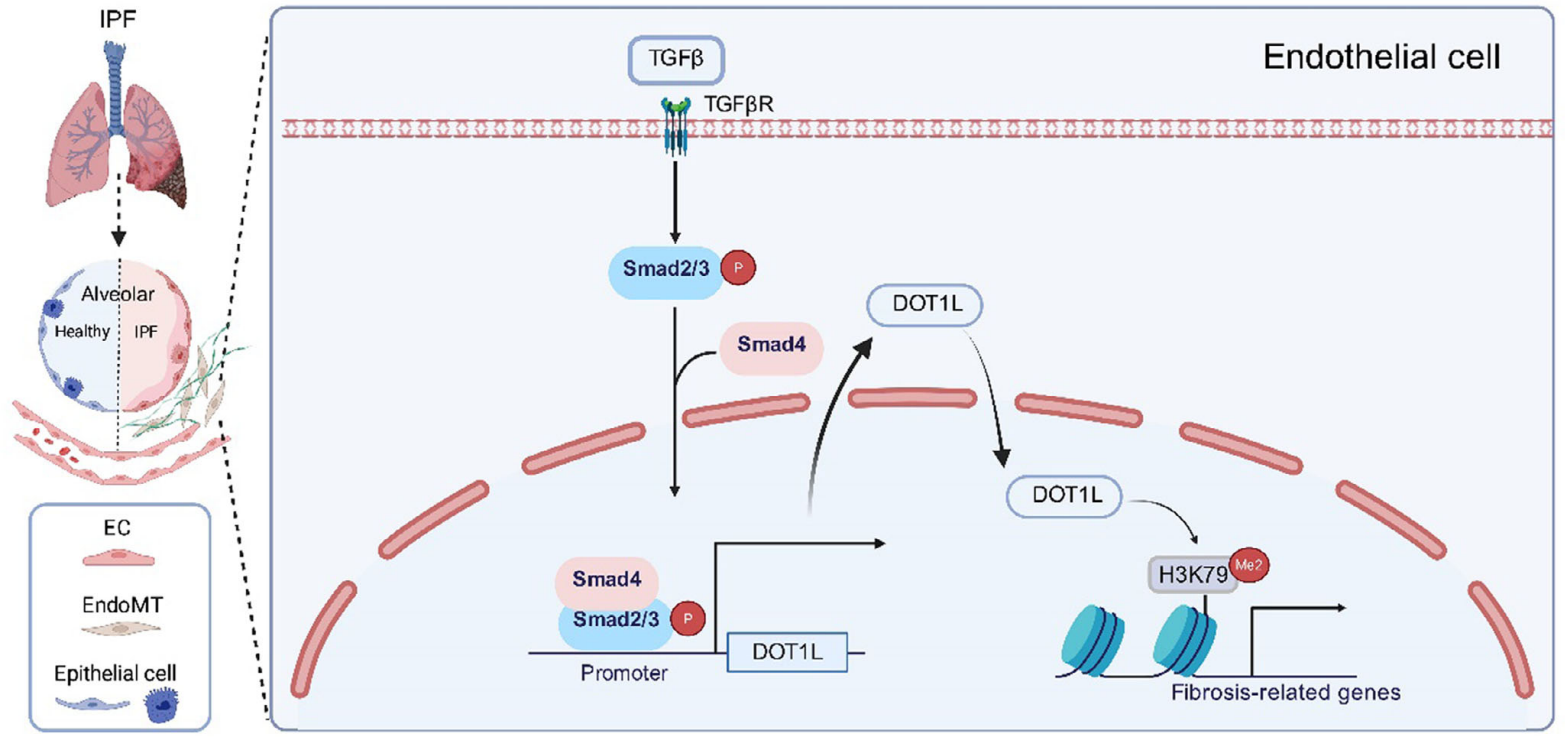
Schematic model of DOT1L in driving EndoMT and pulmonary fibrosis.

In summary, our study identifies DOT1L as a central epigenetic regulator of EndoMT and pulmonary fibrosis. DOT1L functions as an early epigenetic switch, translating TGFβ–SMAD signaling into H3K79me2‐mediated chromatin remodeling, selectively activating fibrosis‐associated genes, and priming ECs for rapid mesenchymal transition (Figure 8). These findings illuminate the molecular underpinnings of EndoMT and position DOT1L as a compelling therapeutic target in IPF and other fibrotic diseases. Moreover, DOT1L‐mediated chromatin remodeling likely represents a broader mechanism enabling cellular plasticity during transdifferentiation and nuclear reprogramming.

## 4 Methods

### 4.1 Cell Culture

Human umbilical vein ECs (HUVECs; ATAC, PCS‐100‐010) were cultured in endothelial growth medium (EGM2; Lonza, cc‐3162) in a humidified incubator at 37°C with 5% CO_2_. For in vitro induction of EndoMT, HUVECs were treated with 10 ng/ml TGFβ2 (PeproTech, 100‐35B) every other day. Cells were collected at the indicated time points for downstream analyses. TIME cells, the immortalized endothelial cell lines, were also cultured in EGM2 medium.

siRNA transfection: HUVECs at approximately 80% confluence were transfected with a 10 nm siRNA pool (Ribbio) using Lipofectamine RNAiMAX (Thermo Fisher, 13778100) in Opti‐MEM medium (Thermo Fisher, 31985070) for 6 h. The transfection medium was then replaced with complete EGM‐2 medium, and cells were cultured for an additional 48 h. The sequences of the siRNAs used are provided in Table S1.

### 4.2 Animal Care and Use

All relevant procedures involving animal experiments presented in this study are compliant with ethical regulations regarding animal research and were conducted under the approval of the Animal Care and Use Committee of the Guangzhou National Laboratory.

### 4.3 Mice

All mice were maintained under specific pathogen‐free conditions in the central animal facility of the Guangzhou National Laboratory. Male mice aged 10–14 weeks were used in all experiments.

Cdh5‐creERT2 mice [51] were used for conditional expression of Cre recombinase. Rosa26‐tdTomato mice [52] express tdTomato after recombination. Dot1L^f/f^ mice on a C57BL/6 background were generated by constitutive floxing of exon 2 of Dot1L gene. This line was established by GemPharmatech (Nanjing, China) via pronuclear microinjection of mouse zygotes with Cas9, guide RNA (gRNA), and donor DNA. sgRNA sequences are provided in Table S2. Microinjected zygotes were transferred into pseudo‐pregnant female recipients, and offspring were genotyped to identify founders carrying floxed Dot1L alleles.

To generate endothelial‐specific inducible knockout mice, Dot1L^f/f^ mice were crossed with Cdh5‐creERT2 mice, producing Cdh5‐creERT2; Dot1L^f/f^ progeny. Cre‐mediated recombination was induced by intraperitoneal injection of tamoxifen (Sigma–Aldrich, USA) at 100 mg/kg body weight once daily for five consecutive days. Mice were used for experiments seven days after the final injection.

### 4.4 Genotyping

Genomic DNA was extracted from mouse tail tips, and genotyping was performed using PCR. Primer sequences are listed in Table S3.

Bleomycin‐induced pulmonary fibrosis model: Ten‐ to twelve‐week‐old male mice were anesthetized and administered intratracheally with 5 U/kg bleomycin (Selleck, S1214).

### 4.5 Real‐Time PCR (RT‐qPCR) and RT‐qPCR Array

Total RNA was extracted using the FastPure Cell/Tissue Total RNA Isolation Kit V2 (Vazyme, RC112‐01), and reverse transcribed into cDNA using the HiScript III RT SuperMix for qPCR (Vazyme, R323). RT‐qPCR was performed using SYBR Green master mix on a QuantStudio 3 machine (Thermo Fisher) [53]. Gene expression was analyzed using the ΔΔCt method and normalized to housekeeping genes. Primer sequences are listed in Table S4.

For RT‐qPCR array screening, the RT^2^ Profiler PCR Array Human Epigenetic Chromatin Modification Enzymes (Qiagen, PAHS‐085Z) was used according to the manufacturer's instructions.

### 4.6 RNA‐sequencing (RNA‐seq) and Data Analysis

2 µg of total RNA was used to construct cDNA libraries. Mouse libraries were prepared using the NEBNext Ultra RNA Library Prep Kit for Illumina (NEB, E7530L) and sequenced on the NovaSeq 6000 platform (Illumina) to generate 150 bp paired‐end reads. Human libraries were prepared using the VAHTS Universal V6 RNA‐seq Library Prep Kit for Illumina (Vazyme, NR604) and sequenced on the Nova X PLUS platform (Illumina), also generating 150 bp paired‐end reads.

Raw sequencing reads were processed for quality control using TrimGalore (v0.6.10; https://github.com/FelixKrueger/TrimGalore) to remove adaptor sequences and low‐quality reads. Clean reads were aligned to either the human reference genome (hg38) using STAR (v2.7.10b) [54] or the mouse reference genome (mm10) using HISAT2 (v2.1.0) [55]. Multi‐mapped reads were excluded. Gene‐level read counts were quantified using featureCounts (v2.0.6) [56] for human samples and HTSeq (v0.6.0) [57] for mouse samples.

Differential gene expression analysis was performed using the DESeq2 package (v1.40.2), with significance thresholds set at adjusted p‐value < 0.05 and fold change > 1.2 for human data and > 1.5 for mouse data [58]. Gene Ontology (GO) and Gene Set Enrichment Analysis (GSEA) were conducted using the clusterProfiler package (v4.9.0). Expression heatmaps were generated using the pheatmap package (v1.0.12; https://github.com/raivokolde/pheatmap).

### 4.7 Western Blot

Samples were collected and lysed in the RIPA buffer containing protease inhibitor cocktail [59]. Protein samples were separated on 4%–20% gradient SDS‐PAGE gels (Beyotime, D0186S) and transferred to the nitrocellulose membrane (Millipore, HATF00010). Blots were blocked at room temperature in 5% non‐fat milk for 1 h and incubated overnight at 4°C with primary antibodies: DOT1L (Abcam, ab239358), H3K79me2 (EpigenTek, C10009‐1), H3K79me1 (EpigenTek, C10009‐1), H3K79me3 (EpigenTek, C10009‐1), SMAD2 (Abclonal, A19114), SMAD3 (Abclonal, A19115), SMAD4 (Abclonal, A19116), p‐SMAD2 (Cell Signaling Technology, CST, 18338), p‐SMAD3 (CST, 9520), SNAI1 (Abclonal, A11794), Fibronectin (Proteintech, 156131‐1‐AP), Vimentin (Abcam, ab20346), α‐SMA (Merck, A2547), β‐Tubulin (Abcam, ab0046), β‐Actin (Abcam, ab8227), GAPDH (Abcam, ab9482) and H3 (CST, 4620s). Blots were then incubated with HRP‐conjugated secondary antibodies for 1 h at room temperature. Signal was detected using ECL reagent and visualized on a MinChemi 610 chemiluminescent imaging system.

Chromatin Immunoprecipitation (ChIP): ChIP assay was performed using the SimpleChIP Enzymatic chromatin IP Kit (CST, 9003S) as previously described [60]. Briefly, HUVECs (∼1 × 10^7^ cells per sample) were collected and cross‐linked with 1% formaldehyde for 10 min at room temperature, then quenched with 125 mm glycine to terminate cross‐linking. Cells were washed and resuspended in 8 mL of Buffer A, then centrifuged at 3000 rpm for 5 min at 4°C; the pellet was resuspended in Buffer B and subjected to a second centrifugation. Nuclei were then washed twice with 1 mL of ChIP buffer and sonicated for 10 min using a Covaris S220 sonicator. Chromatin (10 µg per reaction) was incubated with the pre‐blocked protein G magnetic beads in ChIP buffer containing protease inhibitors for 1 h at 4°C. Immunoprecipitation was performed overnight at 4°C with anti‐H3K79me2 (Abcam, ab3594), anti‐SMAD2/3 (Abcam, ab202445), or anti‐SMAD2 (CST, 5539) antibodies. The following day, immune complexes were captured using protein G magnetic beads, washed three times with ChIP buffer, and once with ChIP buffer containing 0.3 m NaCl. Chromatin was eluted in 150 µL elution buffer at 65°C for 30 min with shaking (1200 rpm). Cross‐linking was reversed by RNase A treatment at 37°C for 2 h, followed by proteinase K digestion at 65°C overnight. ChIP‐qPCR or PCR was performed using primers listed in Table S5.

### 4.8 ChIP‐sequencing (ChIP‐seq) Library Preparation and Data Analysis

For ChIP‐seq, the above purified DNA was end‐repaired, A‐tailed, and ligated with Illumina sequencing adaptors using the NEBNext Ultra DNA Library Prep Kit (NEB, E7645B). Libraries were assessed for quality by Annoroad and sequenced on the Illumina NovaSeq X PLUS platform to generate 150‐bp paired‐end reads.

Raw ChIP‐seq reads were subjected to quality control as described in RNA‐seq data. Clean reads were aligned to the human reference genome (hg38) using Bowtie2 (v2.5.1, –maxins 700, –no‐unal, –no‐mixed, –no‐discordant) [61]. Reads mapping to the mitochondrial genome or unlocalized scaffolds were discarded. Only uniquely mapped reads with a mapping quality ≥ 30 and non‐duplicated reads were finally retained. Peak calling was performed using MACS2 (v2.2.8) with the following parameters: –nomodel, –keep‐dup all, –gsize hs, –broad, –broad‐cutoff 0.01, –qvalue 0.01) [62]. DiffBind package (v3.14.0) [63] was used for differentially binding analysis (fold change > 1.2 and p value < 0.05). ChIPseeker (v1.37.0) [64] was used for peak annotation. Coverage tracks with replicates merged for each group were generated using the bamCoverage or bamCompare function in deepTools (v3.5.2) [65], and visualized with Integrative Genomics Viewer (IGV). ChIP‐seq signal enrichment around transcription start sites (TSS) and across gene bodies was computed using the computeMatrix function in deepTools.

### 4.9 Luciferase Reporter Assay

The DOT1L wild‐type promoter (−500 bp to +100 bp), which contains the SMAD2 binding motif (CCGGAC), was cloned into the pGL3‐enhancer plasmid. Mutant promoter constructs were generated by deleting the SMAD2 binding sequence (Mut1) or by introducing point mutations that changed CCGGAC to CCGAGT (Mut2) or CCGGGT (Mut3).

TIME cells were transfected with these reporter plasmids along with the pRL‐TK Renilla plasmid as an internal control. After 24 h, cells were treated with TGFβ2 for 4 h and then lysed using Passive Lysis Buffer (Promega). Luciferase activity was quantified on a BioTek SYNERGY NEO2 plate reader using the Dual‐Luciferase Reporter Assay System (Promega).

### 4.10 Histology

For histological analysis, lung tissues were inflated and fixed in 4% paraformaldehyde (PFA) at room temperature overnight. Samples were then dehydrated, embedded in paraffin, and sectioned at 4 µm. To assess lung architecture and fibrotic changes, tissue sections were stained with hematoxylin and eosin (H&E) and Masson's Trichrome. Images were analyzed using OlyVIA 4.1 (Olympus, Japan) and ImageJ (National Institutes of Health, USA).

### 4.11 Immunofluorescence (IF) Staining

Paraffin‐embedded sections were deparaffinized in xylene, rehydrated through graded ethanol, and subjected to antigen retrieval in Tris‐EDTA buffer (pH 9.0) using a microwave. Sections were blocked with 5% donkey serum for 30 min at room temperature, and incubated overnight at 4 °C with primary antibodies diluted in 1% donkey serum. After three PBS washes, sections were incubated with fluorophore‐conjugated secondary antibodies for 1 h, washed three additional times with PBS, and counterstained with DAPI for 5 min. Slides were mounted with anti‐fade medium and imaged using a Zeiss LSM 800 confocal microscope. Images were analyzed with ImageJ.

### 4.12 Lung Tissue Dissociation and Flow Cytometry

Mouse lung tissues were collected into gentleMACS C Tubes (Miltenyi Biotec, 130‐093‐237) and incubated in digestion buffer containing 5 mg/mL collagenase I (Worthington, LS004188) and 1 mg/mL DNase I (Worthington, LS002147) in DMEM medium. Tissue dissociation was performed using the gentleMACS Octo Dissociator according to the manufacturer's instructions. The resulting cell suspension was sequentially filtered through 100 and 40 µm strainers to remove debris, then resuspended in FACS buffer (PBS with 2% fetal bovine serum).

For flow cytometry analysis, the single‐cell suspensions were incubated with a Fc‐blocker in FACS buffer on ice for 15 min, centrifuged at 1500 rpm for 5 min at 4°C, and then stained with antibodies on ice for 30 min. After two washes, cells were resuspended in FACS buffer and analyzed on a Beckman CytoFLEX S.

For FACS sorting of ECs, single‐cell suspensions from mouse lung tissues were incubated with a Fc blocker, then stained with CD45 and CD144 on ice for 30 min, followed by DAPI staining. DAPI^−^CD45^−^CD144^+^ cells were sorted using a BD FACSAria Fusion.

### 4.13 Micro‐CT

Mice were anesthetized and scanned using a Quantum GX2 micro‐CT system at 90 kV and 88 µA for 4 min.

### 4.14 ScRNA‐seq Data Analysis

To examine human DOT1L expression across lung endothelial subtypes, we analyzed the public scRNA‐seq dataset GSE136831 [29], which includes 28 healthy controls and 32 idiopathic pulmonary fibrosis (IPF) patients. Raw count matrices and cell annotation files were downloaded from the Gene Expression Omnibus (https://www.ncbi.nlm.nih.gov/geo/). ECs were extracted and analyzed using the Seurat package (v4.3.0), including quality control, normalization, scaling, dimensionality reduction, and visualization via uniform manifold approximation and projection (UMAP). DOT1L expression in different endothelial subtypes was calculated using the AverageExpression function in Seurat. Only patient samples with ≥5 cells in aerocyte or arterial EC populations were included for expression comparisons.

### 4.15 Statistical Analysis

Statistical analysis was performed using GraphPad Prism 9.5. All data were expressed as the mean ± S.E.M. All in vitro experiments in this study were performed with at least three biological replicates, and each experiment was independently repeated a minimum of three times. A one‐way ANOVA followed by Tukey multiple comparisons was used to analyze multiple groups. ^*^ *P*<0.05, ^**^ *P*<0.01, and ^***^ *P*<0.001 were considered statistically significant.

## Conflicts of Interest

The authors declare no conflicts of interest.

## Supporting information




**Supporting File**: advs75117‐sup‐0001‐SuppMat.docx.

## Data Availability

All sequencing data generated in this study have been deposited on NCBI GEO database. Human RNA‐seq data are available under accession number GSE297646; human ChIP‐seq data for H3K79me2 under GSE297647, and for SMAD2/3 under GSE299484. Mouse RNA‐seq data are available under accession number GSE289939. Other data that support the findings of this study are available on request from the corresponding author.
