# Supplementary material for: DOT1L Drives Endothelial‐to‐Mesenchymal Transition and Fibrotic Vascular Remodeling via H3K79 Methylation
Source: Adv Sci (Weinh). 2026 Apr 3;13(34):e15581. doi: 10.1002/advs.202515581 (PMC13285118; doi:10.1002/advs.202515581)
Supplement: Supplementary file 1 — Supporting File: advs75117‐sup‐0001‐SuppMat.docx. [file ADVS-13-e15581-s001.docx]

**DOT1L drives endothelial-to-mesenchymal transition and fibrotic vascular remodeling via H3K79 methylation**

Yaofeng Wang^1,2^, Xing Peng^1^, Jingjing Chen^1,3^, Yun Zhang^1, 4^, Tinghong Zhang^1^, Jingyuan Zhang^1^, Jiaying Fan^1,3^, Hui Zheng^3^, Qiaoyuan Liu^1^, Zhimin Song^1*^, Zhan-Peng Huang^4,5^, Shu Meng^1*^

^1^ Department of Basic Science Research, Guangzhou National Laboratory, Guangzhou, Guangdong, 510005, China

^2^ Zhongshan school of medicine, Sun Yat-sen University, Guangzhou, Guangdong, 510080, China

^3^ State Key Laboratory of Respiratory Disease, the First Affiliated Hospital, Guangzhou Medical University, Guangzhou, Guangdong, 510120, China

^4^ Department of Cardiology, Center for Translational Medicine of Precision Medicine, The First Affiliated Hospital, Sun Yat-sen University, Guangzhou, Guangdong, 510080, China

^5^ NHC Key Laboratory of Assisted Circulation, Sun Yat-sen University, Guangzhou, Guangdong, 510080, China

* Corresponding author:

Zhimin Song, PhD

Address: 9th floor, Building C, No.9, Xing Dao Huan Bei Road, Guangzhou International Bio Island, Haizhu District, Guangzhou, Guangdong Province, China

E-mail: song_zhimin@gzlab.ac.cn

Shu Meng, MD, PhD

Address: 9th floor, Building C, No.9, Xing Dao Huan Bei Road, Guangzhou International Bio Island, Haizhu District, Guangzhou, Guangdong Province, China

E-mail: [meng_shu@gzlab.ac.cn](mailto:meng_shu@gzlab.ac.cn)

**Supplemental figure**


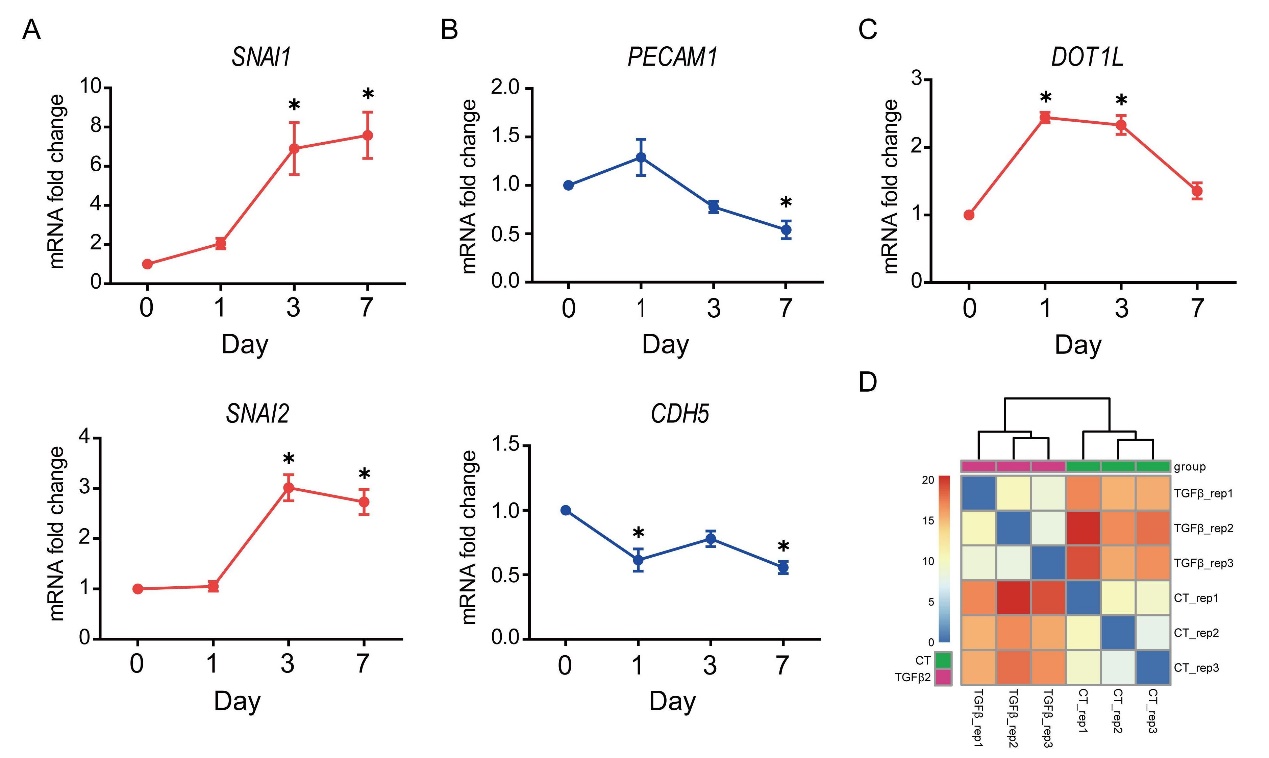


**Supplemental Figure 1. TGFβ2 enhances DOT1L expression and induces EndoMT.**

HUVECs were treated with 10 ng/ml TGFβ2 every other day for 7 days. A. RT-qPCR analysis of mesenchymal markers (*SNAI1* and *SNAI2*). B. RT-qPCR analysis of endothelial markers (*PECAM1* and *CDH5*). C. RT-qPCR analysis of *DOT1L*. D. Spearman analysis of RNA-seq. Data are presented as mean± S.E.M. **p* < 0.05.


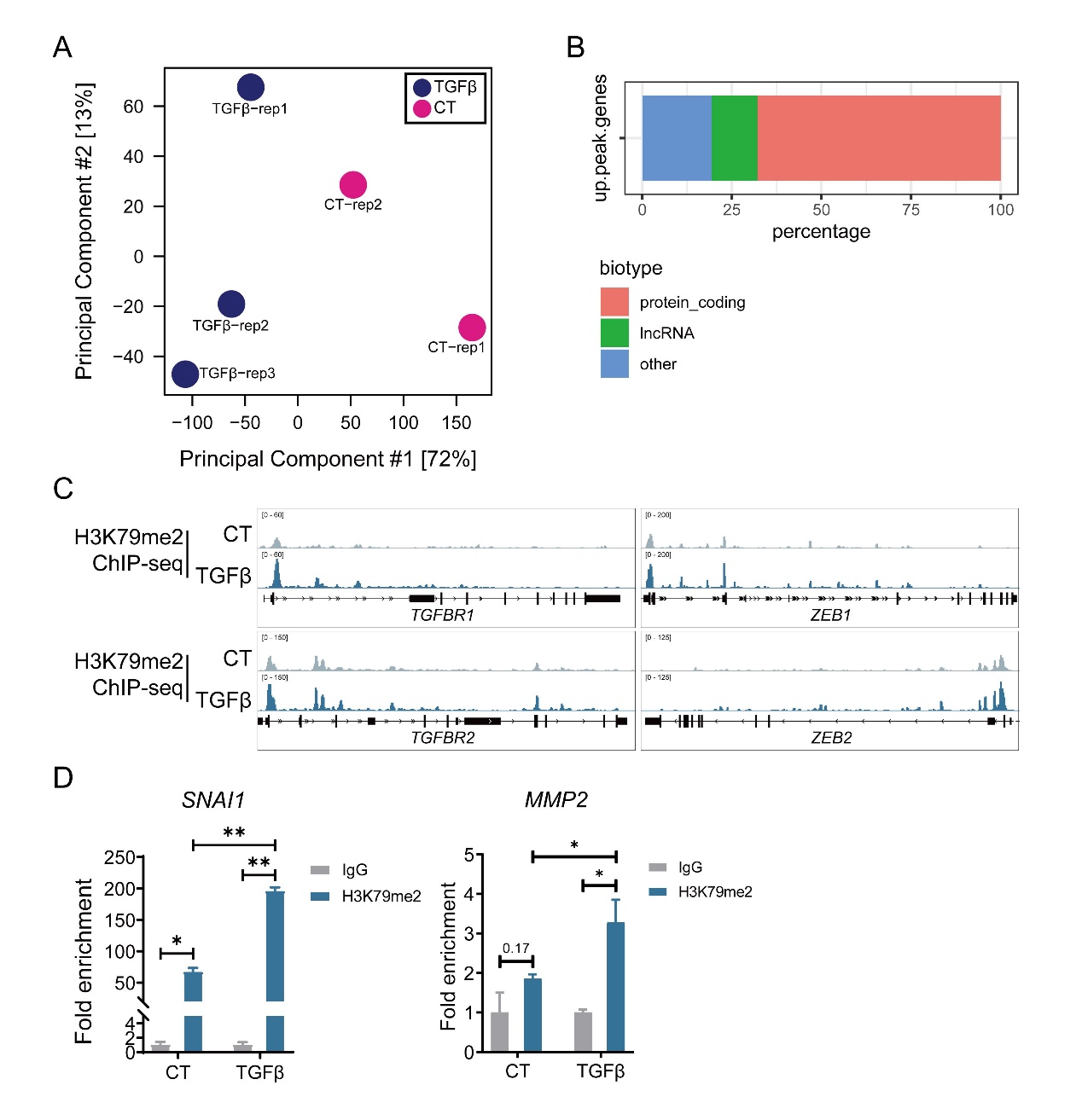


**Supplemental Figure 2. ChIP-seq of TGFβ2-induced EndoMT in HUVECs.**

HUVECs were treated with 10 ng/ml TGFβ2 for 24h. A. Principal component analysis of ChIP-seq data. B. Biotype distribution of genes with increased H3K79me2 peaks. C. ChIP-seq signal of H3K79me2 at the genomic loci of *TGFBR1*, *TGFBR2*, *ZEB1*, and *ZEB2* with or without TGFβ2 treatment. D. ChIP-qPCR analysis of H3K79me2 occupancy in *SNAI1* and *MMP2* promoters. Data are presented as mean± S.E.M. **p* < 0.05, ***p* < 0.01.


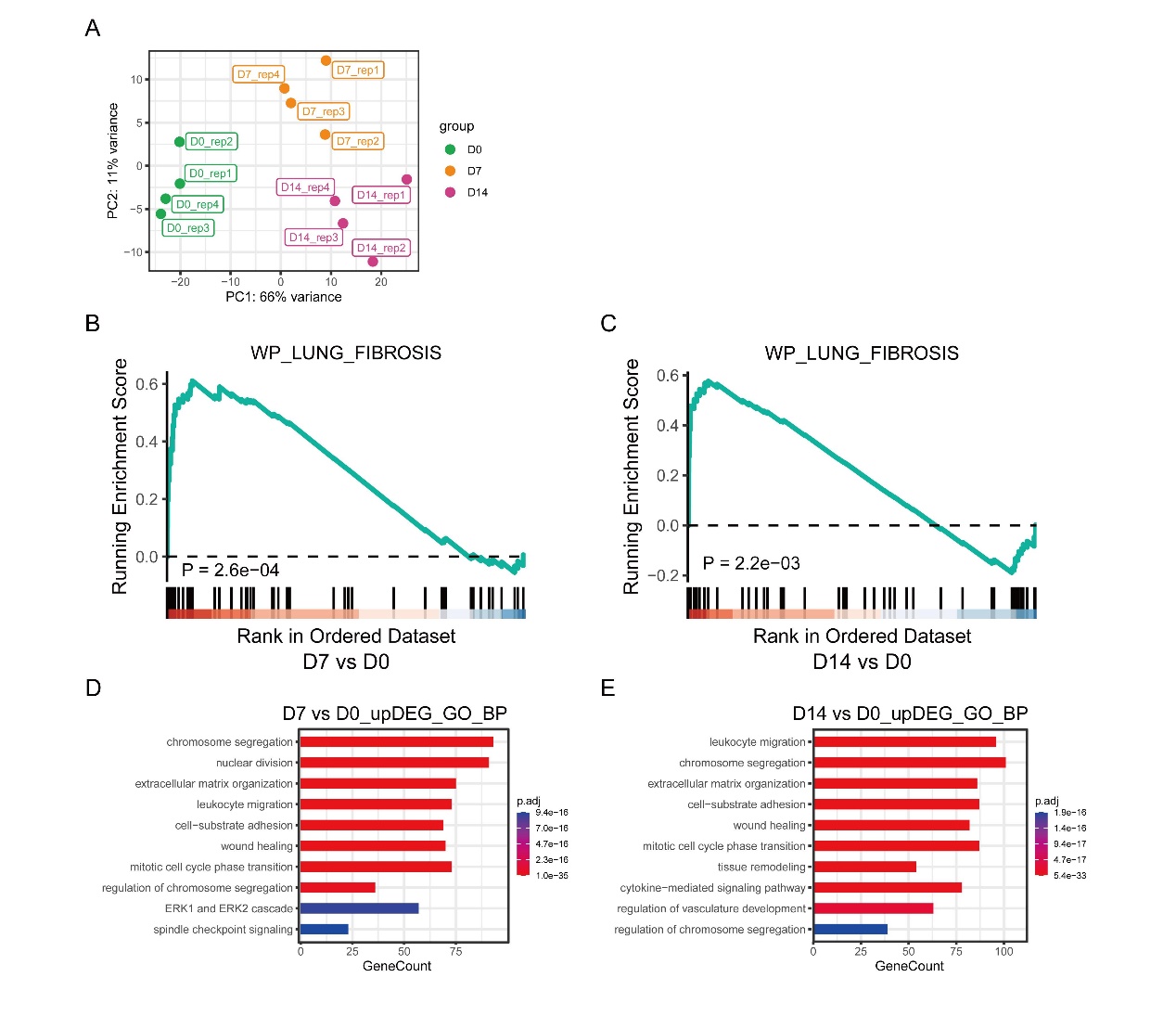


**Supplemental Figure 3. RNA-seq of BLM-induced pulmonary fibrosis.**

A. Principal component analysis showing the correlation between biological replicates of RNA-seq samples. B. GSEA enrichment analysis of lung fibrosis in BLM-induced day 7, compared to day 0. C. GSEA enrichment analysis of lung fibrosis in BLM-induced day 14, compared to day 0. D. GO analysis of the upregulated DEGs in BLM-induced day 7, compared to day 0. E. GO analysis of the upregulated DEGs in BLM-induced day 14, compared to day 0.


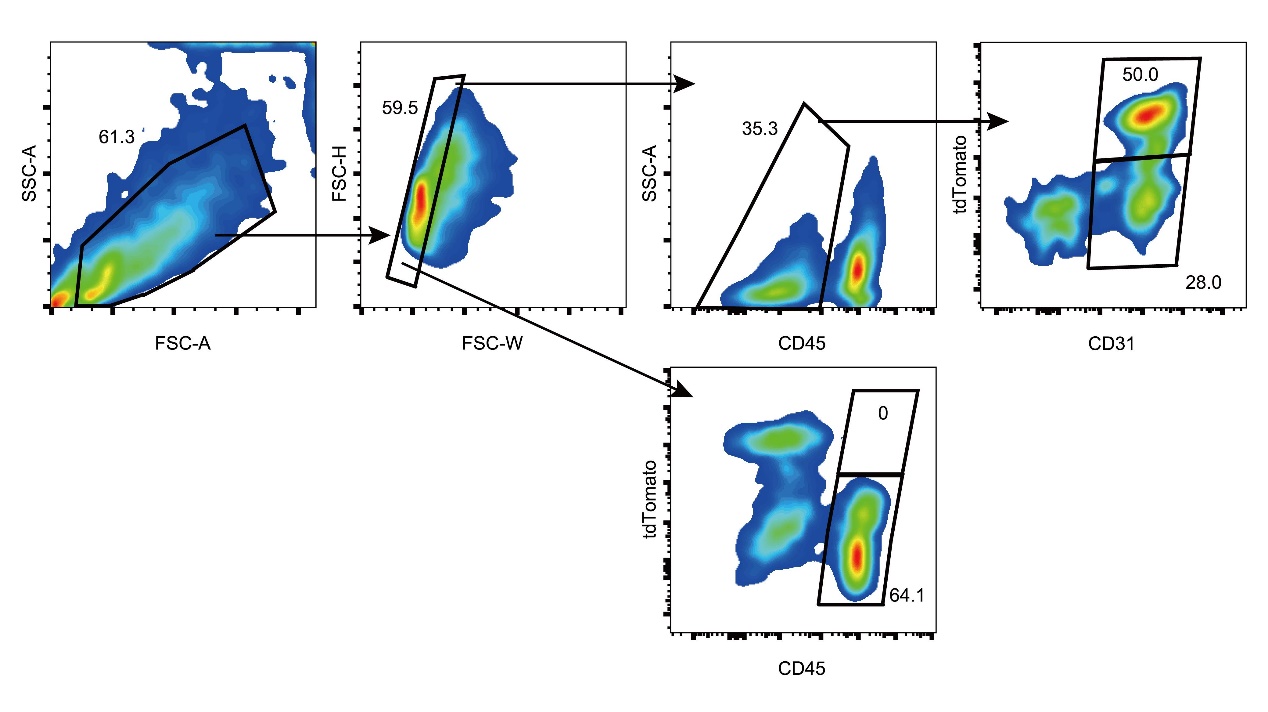


**Supplemental Figure 4. Flow cytometry analysis of lung ECs using endothelial lineage tracing mouse.**

tdTomato was induced by intraperitoneal administration of tamoxifen for 5 days in Cdh5-creERT2; Rosa26-tdTomato mice. Flow cytometry showing tdTomato^+^ cells in CD45⁻CD31⁺ ECs.


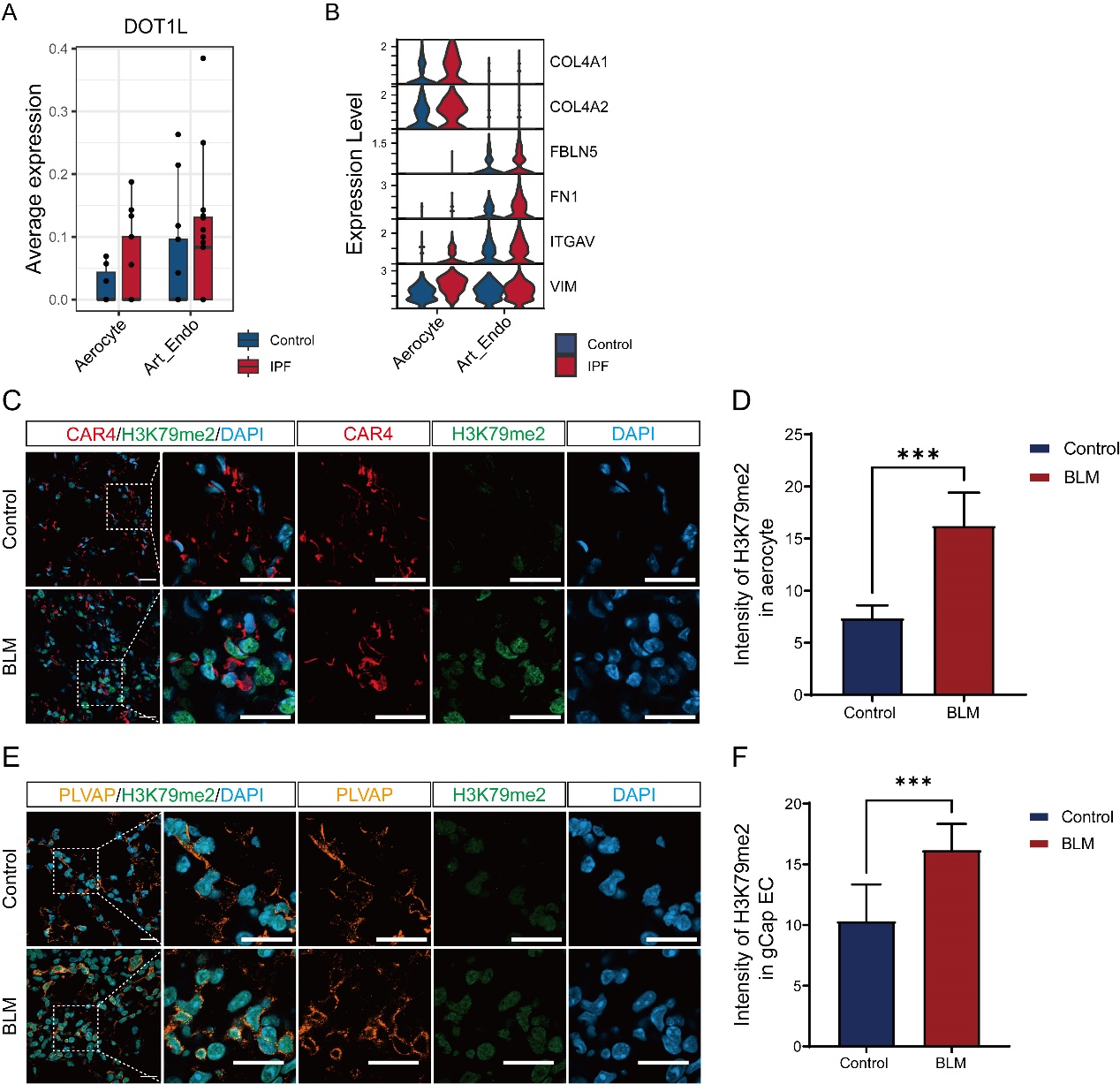


**Supplemental Figure 5. DOT1L expression is upregulated in arterial ECs and aerocytes in human IPF.**

A. Average DOT1L expression in aerocytes and arterial ECs from GSE136831. B. Violin plots showing expression of fibrosis-related genes in aerocytes and arterial ECs. C. IF staining of H3K79me2 in CAR4^+^ aerocytes in lung tissue at day 14 post-BLM. Scale bar: 20 μm. D. H3K79me2 intensity in CAR4^+^ aerocytes. E. IF staining of H3K79me2 in PLVAP^+^ gCap ECs in lung tissue at day 14 post-BLM. F. H3K79me2 intensity in PLVAP^+^ gCap ECs. Data are presented as mean± S.E.M. ****p* < 0.001.


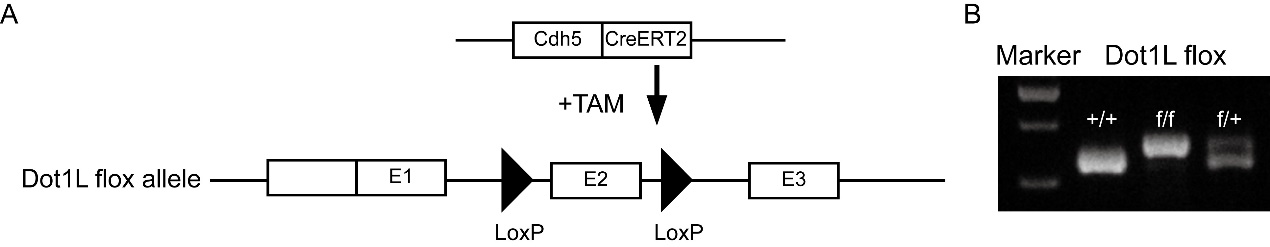


**Supplemental Figure 6. Generation of DOT1L-ECKO mice.**

A. Schematic of the generation of DOT1L-ECKO mice. B. Gel electrophoresis of DOT1L flox genotyping.


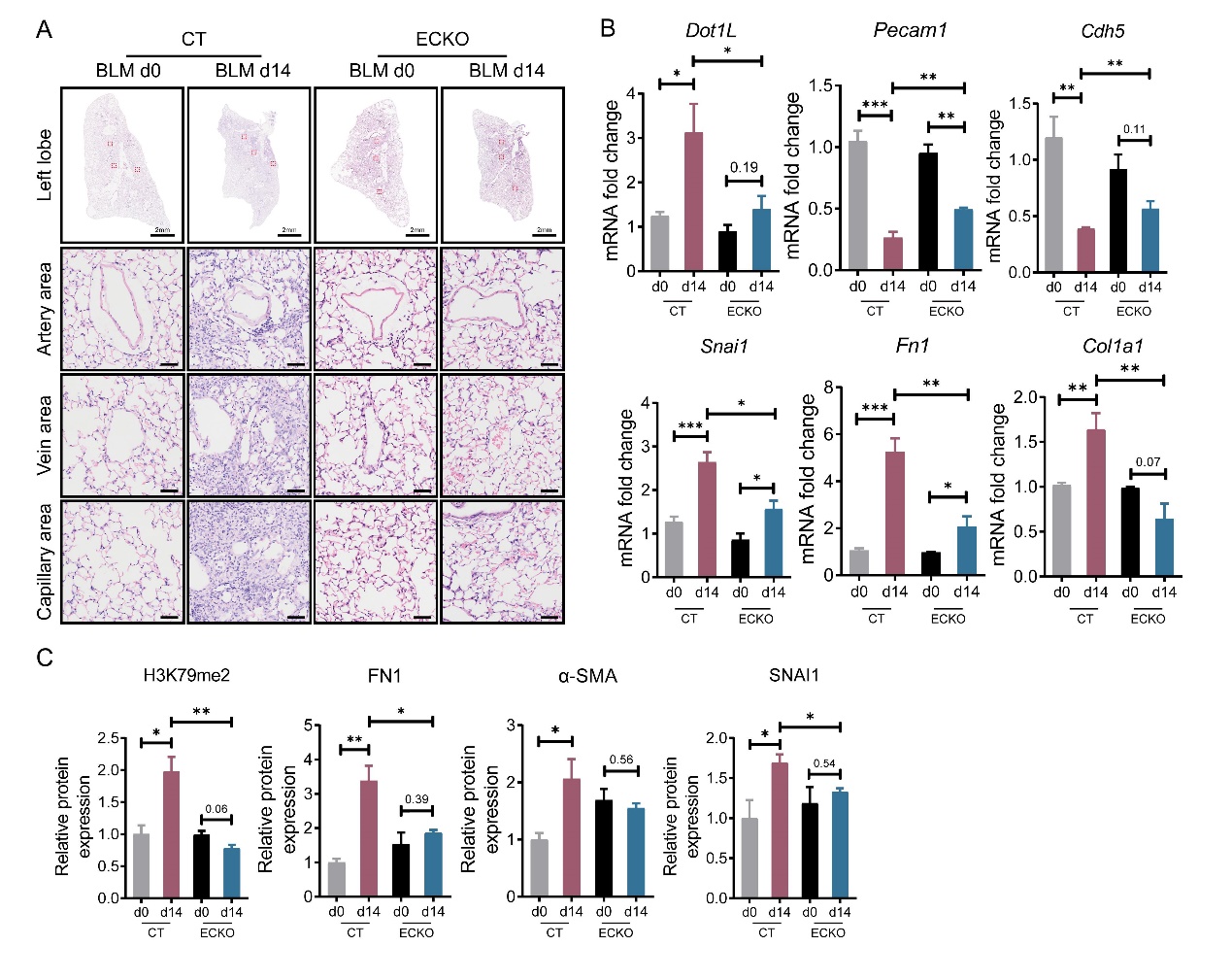


**Supplemental Figure 7. Endothelial-specific Dot1L knockout mitigates pulmonary fibrosis.**

A. H&E staining. Scale bar: 2mm and 50 μm. B. RT-qPCR analysis of lung tissue. C. Quantification of Fig. 7E. Data are presented as mean± S.E.M. **p* < 0.05, ***p* < 0.01, ****p* < 0.001.

**Supplementary table**

**Table 1. siRNA sequences**

| DOT1L siRNA | 5'-3' sequence |
| --- | --- |
| 1 | ACCUCUGAACUUCAGAAUAAACAGT |
| 2 | UAAAAGGCAACUUAUUGAGAAAUAT |
| 3 | CCAACUGCAAACAUCACUAUGGCGT |

**Table 2. sgRNA sequences**

| Dot1L^f/f^ mice sgRNA | 5'-3' sequence | PAM |
| --- | --- | --- |
| 5S1 | GAGCCCATATTAGTGTTCAA | GGG |
| 3S1 | GGCCTCTTTACGGCCCCGGC | TGG |

**Table 3. Genotyping primer sequences**

|  | Forward primer (5'-3') | Reverse primer (5'-3') |
| --- | --- | --- |
| Cdh5-creERT2-wt | AGTGGCCTCTTCCAGAAATG | TGCGACTG GTCTGATTTCC |
| Cdh5-creERT2-mutant | TGGATAGTGAAACAGGGGCAATG | ATAGAGTATGGGGGGCTCAGCATC |
| Rosa26-tdTomato-wt | AAGGGAGCTGCAGTGGAGTA | CCGAAAATCTGTGGGAAGTC |
| Rosa26-tdTomato-mutant | GGCATTAAAGCAGCGTATCC | CTGTTCCTGTACGGCATGG |
| Dot1L^f/f^ | ACATGCACTTCCTCAGCTCAAAC | CTGAAGCATCCTGTGGTTCCTG |

**Table 4. RT-qPCR primer sequences**

| Species | Gene | Forward primer (5'-3') | Reverse primer (5'-3') |
| --- | --- | --- | --- |
| Human | DOT1L | GCCAAGTATGCGGAGACCAT | CAGCTGGTGATCCACCTCAG |
|  | CDH5 | TACCACCTCACTGCTGTCATT | ATTGAACAACCGATGCGTGAA |
|  | PECAM1 | ACGTGCAGTACACGGAAGTT | GGAGCCTTCCGTTCTAGAGT |
|  | SNAI1 | TGCCCTCAAGATGCACATCCGA | GGGACAGGAGAAGGGCTTCTC |
|  | SNAI2 | AAAAGCCAAACTACAGCGAACT | AGGATCTCTGGTTGTGGTATGA |
|  | COL5A1 | TGACAAGAAGTCCGAAGGGG | CGTCCACATAGGAGAGCAGTT |
|  | COL4A1 | GGGGAGCCTGGTGAGTTTTA | CAATCCTACAGAACCCGGCG |
|  | MMP2 | AGGGAGCGCTACGATGGAG | AGGTGTTCAGGTATTGCACTG |
|  | FBLN5 | TCTTCTCGCCTTCGCATCTC | ATTCGTGCACTGTGCCTGT |
|  | FN1 | CAGACCTATCCAAGCTCAAGT | GGTTTCTGGGTGGGATACTCA |
|  | CAV2 | TACAGCTCTTCATGGACGACG | CGAAGCCCAGCTTGAGAT |
|  | KDR | TATGATGTGGTTCTGAGTCCGT | AGGTCTCGGTTTACAAGTTTCT |
| Mouse | Dot1L | GCGGAACCGTTGGAGGTAAT | TTCACAGTGGCTCCATGTCC |
|  | Cdh5 | CCTGAGGCAATCAACTGTGC | GGAGGAGCTGATCTTGTCCG |
|  | Pecam1 | GGAAGTGTCCTCCCTTGAGC | GGAGCCTTCCGTTCTTAGGG |
|  | Snai1 | GGAGTTGACTACCGACCTTGC | CTGGAAGGTGAACTCCACACAC |
|  | Acta2 | CCAGCCATCTTTCATTGGGATG | TACCCCCTGACAGGACGTTG |
|  | Fn1 | GGCCACCATTACTGGTCTGG | GGAAGGGTAACCAGTTGGGG |
|  | Col1a1 | CGATGGATTCCCGTTCGAGT | CGATCTCGTTGGATCCCTGG |

**Table 5. ChIP primer**

| ChIP | Gene | Forward primer (5'-3') | Reverse primer (5'-3') |
| --- | --- | --- | --- |
| H3K79me2 | SNAI1 | GAGGAGCTCCGCAAGAGG | CACACATCTCGTCTCCCCAC |
|  | MMP2 | GCTACGATGGAGGCGCTAAT | AACTTGATGATGGGCGACGG |
| SMAD2/3 and SMAD2 | DOT1L | CCCAATAAACAGCATTGTTGTCG | TAAAACCAAAAATCCGAATTTCCCC |
